# AMPK antagonizes hepatic glucagon-stimulated cyclic AMP signalling via phosphorylation-induced activation of cyclic nucleotide phosphodiesterase 4B

**DOI:** 10.1038/ncomms10856

**Published:** 2016-03-08

**Authors:** M. Johanns, Y.-C. Lai, M.-F. Hsu, R. Jacobs, D. Vertommen, J. Van Sande, J. E. Dumont, A. Woods, D. Carling, L. Hue, B. Viollet, M Foretz, M H Rider

**Affiliations:** 1 Université catholique de Louvain and de Duve Institute, Avenue Hippocrate, 75, 1200 Brussels, Belgium; 2 Faculté de Médecine, Institut de Recherche Interdisciplinaire en Biologie Humaine et Moléculaire (IRIBHM), Université Libre de Bruxelles (ULB), Route de Lennik, 808, 1070 Brussels, Belgium; 3 Cellular Stress Group, MRC Clinical Sciences Centre, Imperial College London, Hammersmith Hospital, DuCane Road, London W12 0NN, UK; 4 INSERM U1016, Institut Cochin, 75014 Paris, France; 5 CNRS UMR8104, 75014 Paris, France; 6 Université Paris Descartes, Sorbonne Paris Cité, 75014 Paris, France

## Abstract

Biguanides such as metformin have previously been shown to antagonize hepatic glucagon-stimulated cyclic AMP (cAMP) signalling independently of AMP-activated protein kinase (AMPK) via direct inhibition of adenylate cyclase by AMP. Here we show that incubation of hepatocytes with the small-molecule AMPK activator 991 decreases glucagon-stimulated cAMP accumulation, cAMP-dependent protein kinase (PKA) activity and downstream PKA target phosphorylation. Moreover, incubation of hepatocytes with 991 increases the *V*_max_ of cyclic nucleotide phosphodiesterase 4B (PDE4B) without affecting intracellular adenine nucleotide concentrations. The effects of 991 to decrease glucagon-stimulated cAMP concentrations and activate PDE4B are lost in hepatocytes deleted for both catalytic subunits of AMPK. PDE4B is phosphorylated by AMPK at three sites, and by site-directed mutagenesis, Ser304 phosphorylation is important for activation. In conclusion, we provide a new mechanism by which AMPK antagonizes hepatic glucagon signalling via phosphorylation-induced PDE4B activation.

## Abstract (web summary)

The diabetes drug Metformin decreases hepatic glucose production and activates AMP-activated protein kinase (AMPK). Here the authors provide evidence that AMPK activation antagonizes glucagon signalling by activating PDE4B, lowering cAMP levels and decreasing PKA activation.

## Graphical abstract

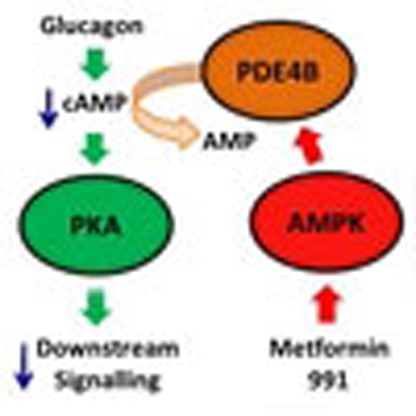

One of the short-term effects of glucagon in liver is to increase glycogen breakdown and inhibit glycolysis^1^,^2^,^3^. Glucagon provides the mechanism for switching the metabolism of liver between the well-fed state and the starved state, which comprises several phases. During the first few hours of starvation, glycogen reserves in the liver are mobilized to maintain blood glucose, but during prolonged starvation, hepatic gluconeogenesis prevails^4^. Glucagon action is mediated by increased cyclic AMP (cAMP) via stimulation of adenylate cyclase and subsequent activation of cAMP-dependent protein kinase (PKA)^5^,^6^. Glycogenolysis is increased via phosphorylase kinase activation downstream of PKA, which also inhibits glycolysis by phosphorylating and inactivating liver 6-phosphofructo-2-kinase/fructose-2,6-bisphosphatase (PFKFB1 isoenzyme), thereby lowering fructose-2,6-bisphosphate concentrations, and by phosphorylation-induced inactivation of L-type pyruvate kinase. In the long-term, glucagon induces the expression of key gluconeogenic enzymes, such as phosphoenolpyruvate carboxykinase and glucose-6-phosphatase^7^,^8^, which together with its short-term effects, accounts for the increase in hepatic gluconeogenesis and glucose output in the fasted state^9^. Type 2 diabetes is characterized by hyperglycaemia and, due to a combination of insulin resistance and impaired β-cell function, the liver excessively releases glucose into the blood. Also, elevated plasma glucagon concentrations play a role in dysregulated hepatic glucose production^10^. Metformin, the front-line drug used worldwide for the treatment of diabetes, decreases hepatic glucose production and activates AMP-activated protein kinase (AMPK)^11^,^12^,^13^,^14^,^15^,^16^. However, whether AMPK and/or its upstream kinase LKB1 is required for the inhibition of hepatic glucose output by biguanides, such as metformin and phenformin, is controversial^17^,^18^,^19^,^20^,^21^,^22^. Biguanides inhibit mitochondrial respiratory chain complex I (ref. 23), resulting in a fall in ATP, and it was proposed that the resulting increase in AMP in hepatocytes would directly inhibit adenylate cyclase to abrogate glucagon-stimulated increases in PKA activity independently of AMPK^21^. Recently, we studied a small-molecule benzimidazole derivative that rapidly and potently activates AMPK^24^, thereby stimulating glucose uptake in skeletal muscle without increasing cellular AMP levels^25^. In the present study, we used the same molecule, referred to as 991, in primary mouse hepatocytes. We propose a mechanism by which AMPK activation antagonizes glucagon signalling by phosphorylating and activating the major hepatic cyclic nucleotide phosphodiesterase (PDE) isoform PDE4B, thereby lowering cAMP levels and decreasing PKA activation.

## Results

### Compound 991 decreases glucagon-stimulated PKA signalling

**Figure 1:**
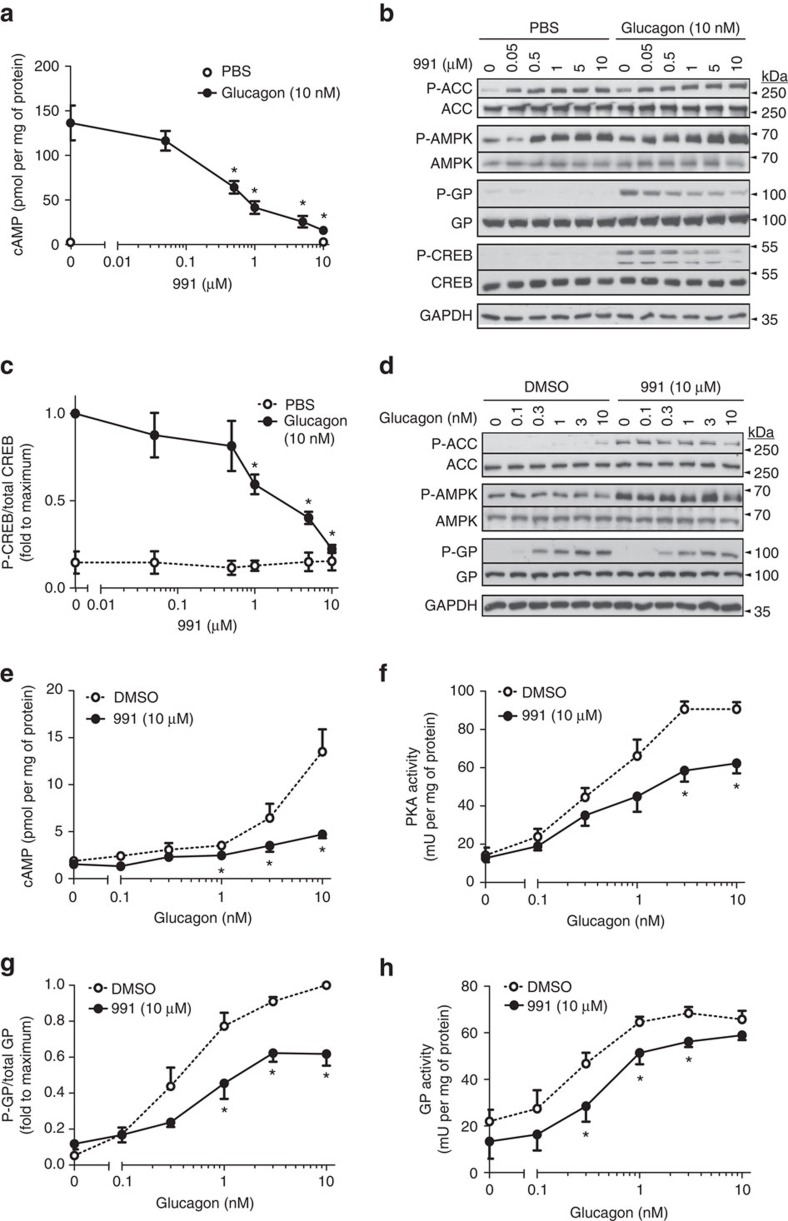
Compound 991 activates AMPK in hepatocytes and decreases glucagon-stimulated PKA signalling. Primary mouse hepatocytes were serum-starved overnight and incubated for 20 min with the indicated concentrations of 991 or dimethylsulfoxide (DMSO) as vehicle before stimulation with the indicated concentrations of glucagon for 15 min. The cells were collected and lysed for the measurement of intracellular cAMP concentrations by radioimmunoassay (**a**) or enzyme-linked immunosorbent assay (ELISA) (**e**), for immunoblotting phosphorylated and total proteins as indicated (**b**,**d**), for PKA assay (**f**), for GP assay (**h**) and for quantification of CREB and GP phosphorylation by immunoblotting (**c**,**g**). Values are means±s.e.m. for *n*=4 (**a**,**f**–**h**) or *n*=5 (**c**,**e**) separate experiments, and in **b** and **d** representative blots are shown. Statistical analysis was by a paired Student's *t*-test. *Indicates a significant difference (*P*<0.05) compared with control incubations with DMSO.

**Figure 2:**
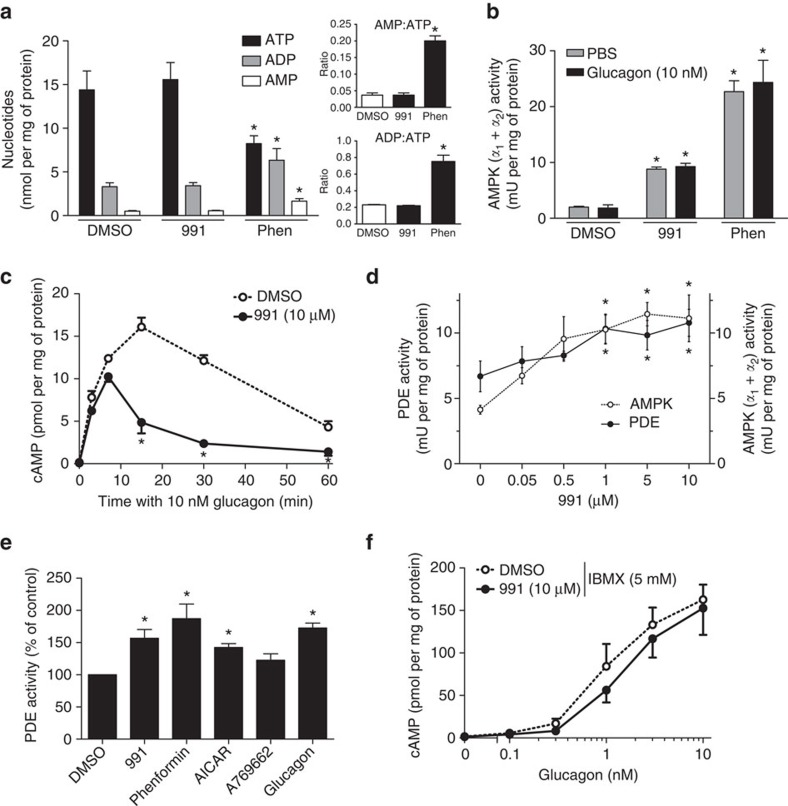
AMPK activation decreases cAMP levels by activating a PDE. Primary mouse hepatocytes were incubated for 1 h with DMSO as vehicle, 10 μM 991, 500 μM phenformin, 2 mM AICAR, 100 μM A769662, 10 nM glucagon or with concentrations of 991 and glucagon as indicated for measurements of intracellular adenine nucleotide concentrations (**a**), AMPK activity by immunoprecipitation using anti-AMPKα1 and anti-AMPKα2 antibodies (**b**,**d**) and total PDE activity (**d**,**e**). In **e**, the 100% value for PDE activity in the DMSO-treated control condition was 6.4±0.7 μU  per mg of protein. In **c**, mouse hepatocytes were incubated for 20 min with 10 μM 991 or DMSO as vehicle before incubation with 10 nM glucagon for ELISA measurements of cAMP concentrations at the indicated times. In **f**, mouse hepatocytes were incubated for 20 min with 10 μM 991 or DMSO as vehicle and 5 mM pan-PDE inhibitor IBMX before incubation with the indicated concentrations of glucagon for 15 min and measurement of cAMP (ELISA method). Values are means±s.e.m. for *n*=3 (**a**–**d**), *n*=5 (**e**) or *n*=4 (**f**) separate experiments. Statistical analysis was by a paired Student's *t*-test. *Indicates a significant difference (*P*<0.05) compared with control incubations with DMSO.

Compound 991 (previously called ex229) is a potent small-molecule AMPK activator that increases glucose uptake in incubated rat and mouse skeletal muscles^25^. In primary mouse hepatocytes, treatment with 991 before incubation with glucagon dose-dependently decreased the glucagon-stimulated increase in intracellular cAMP concentrations (Fig. 1a). Consistent with effects on cAMP levels, 991 treatment antagonized increased phosphorylation by glucagon of PKA downstream targets glycogen phosphorylase (GP)^26^ at Ser14 and cAMP response element-binding protein (CREB)^27^ at Ser133 (Fig. 1b–d,g). Moreover, incubation with a single high dose of 991 antagonized the increases in cAMP levels and PKA activity in response to increasing concentrations of glucagon (Fig. 1e,f). The rise in cAMP levels in response to glucagon is transient (Fig. 2c) and only a small increase in cAMP was needed (Fig. 1e) to elicit PKA activation (Fig. 1f). In hepatocytes preincubated with 991, increased GP activity (Fig. 1h) and increased GP Ser14 phosphorylation induced by glucagon (Fig. 1g) were also reduced.

### AMPK activation leads to increased PDE activity

Direct inhibition of adenylate cyclase by AMP has been proposed to explain how biguanides reduce glucagon-stimulated cAMP production in hepatocytes^21^. Indeed, in hepatocytes incubated with phenformin, a reduction in intracellular ATP levels with concomitant increases in AMP, ADP, AMP:ATP and ADP:ATP ratios were observed (Fig. 2a). Also, treatment with 991 or phenformin led to AMPK activation, which was unaffected by glucagon (Fig. 2b). However, the effects of 991 to activate AMPK and antagonize glucagon action in hepatocytes observed in our study were not accompanied by changes in intracellular adenine nucleotide concentrations, even at the maximal dose of 10 μM (Fig. 2a). A time-course study indicated that there was no significant difference in the initial rate of cAMP production by glucagon in hepatocytes preincubated with 991 (Fig. 2c), suggesting that adenylate cyclase activity was not affected by compound treatment. After 7 min of incubation, glucagon-stimulated cAMP levels decreased rapidly in cells preincubated with 991, such that after 30 min of incubation with glucagon, cAMP concentrations were reduced by 80% (Fig. 2c). We hypothesized therefore, that AMPK activation could lead to an increase in PDE activity, thus lowering cAMP accumulation seen in the presence of glucagon. Indeed, in extracts prepared from hepatocytes incubated with 991, total PDE activity increased by ∼50% in a dose-dependent manner, which correlated with increased AMPK activity (Fig. 2d). Also, PDE activation after treatment with 991 was comparable to that seen in hepatocytes incubated with glucagon (Fig. 2e), which is known to activate PDE as a negative feedback mechanism for cAMP signalling^28^. PDE activation was also observed in hepatocytes incubated with the AMPK activator 5-aminoimidazole-4-carboxamide ribonucleoside (AICAR), but not significantly with A769662, a small-molecule AMPK activator that is not as effective as compound 991, at least in skeletal muscle^25^. Interestingly, treatment of hepatocytes with phenformin increased PDE activity to similar levels to those observed with 991 (Fig. 2e), and incubation with either phenformin or metformin led to dose-dependent PDE activation, which correlated with increased AMPK Thr172 phosphorylation (Supplementary Fig. 1). Moreover, significant increases in PDE activity were obtained with submaximal doses of phenformin (100 μM) and metformin (100–300 μM). To confirm the implication of PDE activity in the effect of 991 to lower cAMP, hepatocytes were incubated with the pan-PDE inhibitor 3-isobutyl-1-methylxanthine (IBMX) with or without 991, before addition of increasing concentrations of glucagon (Fig. 2f). In the presence of IMBX, the effect of 991 to decrease glucagon-induced cAMP accumulation was lost, suggesting PDE dependence. In this experiment, the dose-dependent increase in cAMP levels in response to glucagon was much higher (Fig. 2f versus Fig. 1e), indicating that the PDE inhibitor was effective.

### Effects of compound 991 treatment are AMPK-dependent

**Figure 3:**
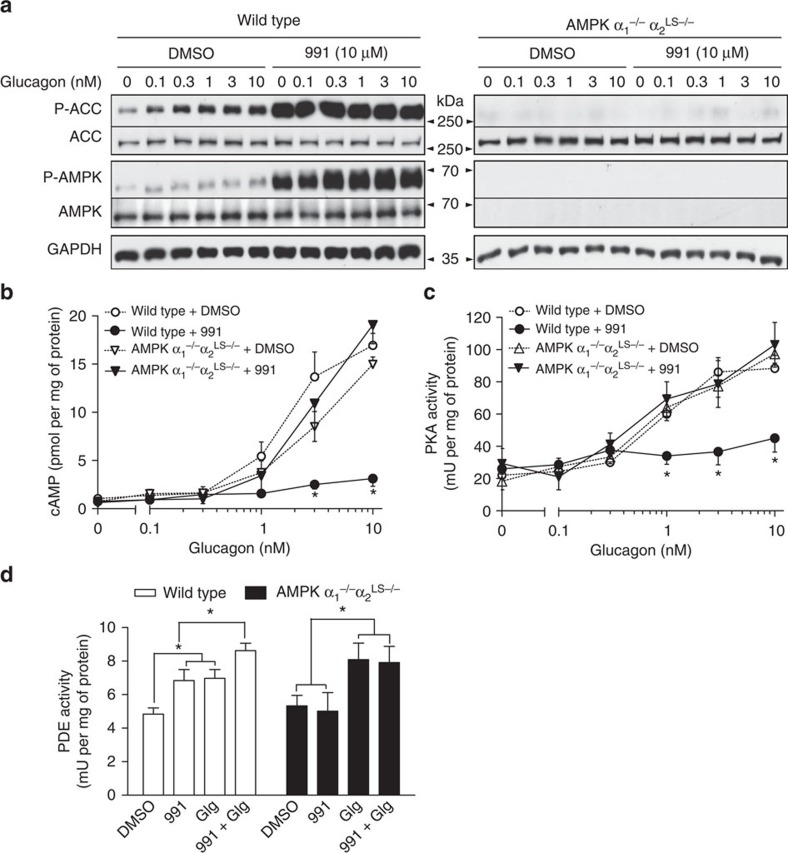
Compound 991 antagonizes glucagon signalling in an AMPK-dependent manner. Primary hepatocytes from wild-type mice or mice bearing a liver-specific deletion of the two AMPK catalytic subunits (AMPK α_1_^−/−^ α_2_^LS−/−^) were treated as described in the legends to Figs 1 and 2. The cells were collected and lysed for immunoblotting levels of phosphorylated ACC and AMPK versus total proteins along with GAPDH as a loading control (**a**). Extracts were also prepared for the measurement of cAMP concentrations by ELISA (**b**), for PKA assay (**c**) and for PDE assay (**d**). Values are means±s.e.m. for *n*=3 (**b**–**d**) separate experiments, and in **a** representative immunoblots are shown. Statistical analysis was by a paired Student's *t*-test. *Indicates a significant difference (*P*<0.05) compared with control incubations with DMSO or between the indicated conditions.

To test whether the effects of compound 991 treatment were AMPK-dependent, primary hepatocytes were isolated from mice in which the two catalytic subunits of AMPK had been specifically deleted in liver (referred to as AMPK α_1_^−/−^α_2_^LS−/−^). In hepatocytes from wild-type mice incubated with increasing concentrations of glucagon, phosphorylation of ACC and AMPK slightly increased, as seen by immunoblotting extracts (Fig. 3a), even though AMPK activation by glucagon was not detected after one hour of incubation (Fig. 2b). However, the effect of glucagon to increase ACC phosphorylation was absent in hepatocytes from AMPK α_1_^−/−^α_2_^LS−/−^ mice (Fig. 3a), suggesting that AMPK mediates this effect. Moreover, the effect of incubation with 991 to increase ACC phosphorylation in hepatocytes from control mice was almost completely lost in hepatocytes from AMPK α_1_^−/−^α_2_^LS−/−^ mice and both total and pThr172-AMPK were undetectable (Fig. 3a). In addition, the effects of 991 to abrogate glucagon-stimulated cAMP accumulation (Fig. 3b) and PKA activation (Fig. 3c) were abolished in incubations of hepatocytes from AMPK α_1_^−/−^α_2_^LS−/−^ mice, indicating the requirement of AMPK. Similar results were obtained in incubations of hepatocytes prepared from mice bearing a whole-body deletion of the AMPK β1-subunit, which contributes towards providing a high-affinity binding site for compound 991 (ref. 24). There is almost no AMPK activity in the liver of these mice (Woods and Carling, unpublished data), and residual AMPK activity might have been due to increased expression of the AMPK β2-subunit (Supplementary Fig. 2B). In hepatocytes from AMPK β1-knockout compared with wild-type mice, the effect of 991 to activate AMPK and increase ACC phosphorylation was severely abrogated and the increase in Raptor (regulatory-associated protein of mTOR) phosphorylation, another direct AMPK substrate, was completely lost (Supplementary Fig. 2B). Importantly, in hepatocytes from AMPK β1-knockout versus wild-type mice, the effect of 991 to lower glucagon-stimulated cAMP accumulation was markedly reduced (Supplementary Fig. 2A). Treatment of hepatocytes from control mice with 991 increased PDE activity in extracts by ∼50%, whereas in hepatocytes from AMPK α_1_^−/−^α_2_^LS−/−^ mice the effect of 991 was lost, further confirming the requirement of AMPK. By contrast, incubation with glucagon led to PDE activation in hepatocytes from both wild-type and AMPK α_1_^−/−^α_2_^LS−/−^ mice (Fig. 3d).

### PDE4 is the major isoenzyme activated by 991 in hepatocytes

**Figure 4:**
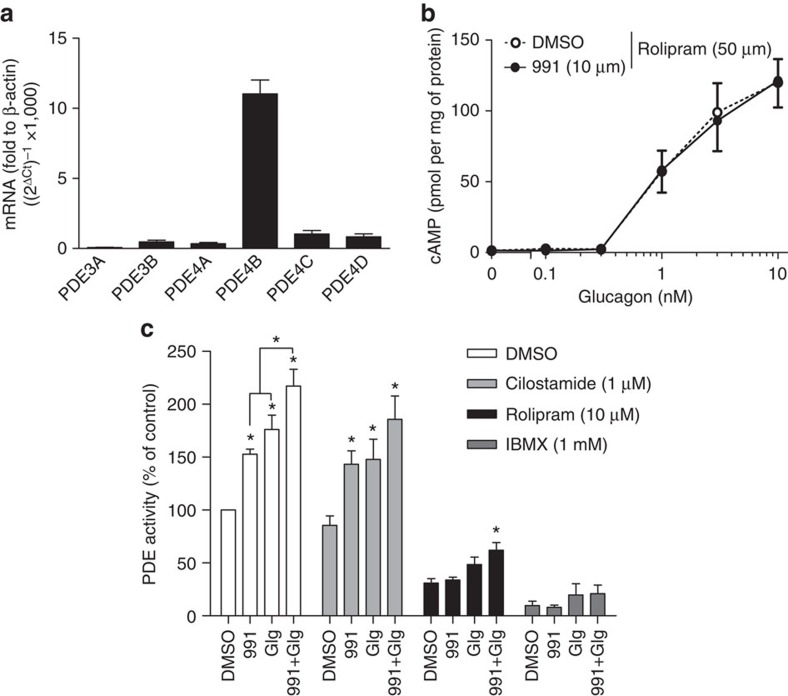
PDE4 is the major isoenzyme in cultured mouse hepatocytes and is activated by treatment with compound 991. Primary mouse hepatocytes were serum-starved overnight, then total RNA was extracted for cDNA synthesis and real-time PCR using specific primers for the mouse PDE isoenzymes indicated (**a**). In **b**, hepatocytes were incubated for 20 min with 991 or with DMSO as vehicle control in the presence of the PDE4 inhibitor rolipram before stimulation with indicated concentrations of glucagon for 15 min. The cells were collected and lysed for the measurement of cAMP concentrations by ELISA. In **c**, the cells were lysed after 1 h of treatment with 991 (10 μM) and/or glucagon (Glg, 10 nM), and extracts were assayed for total PDE activity in the presence or absence of PDE inhibitors as indicated. The 100% value for PDE activity in extracts from control DMSO-treated cells was 2.7±0.7 μU per mg of protein. Values are means±s.e.m. for *n*=3 (**a**), *n*=6 (**b**) or *n*=5 (**c**) separate experiments. Statistical analysis was by a paired Student's *t*-test. *Indicates a significant difference (*P*<0.05) compared with control incubations with DMSO or between the indicated conditions.

The expression of PDE isoenzymes in primary mouse hepatocytes was investigated. Earlier studies suggested that the major PDE isoenzymes expressed in liver are members of the PDE3/4 subfamilies^29^,^30^,^31^. Real-time PCR revealed that amongst these, the predominant PDE mRNA in primary mouse hepatocytes was the PDE4B isoenzyme (Fig. 4a). The implication of PDE isoenzymes in cAMP hydrolysis in hepatocytes was assessed by the use of specific inhibitors *in vitro*. When hepatocytes were preincubated with the PDE4-specific inhibitor rolipram and 991 before addition of increasing concentrations of glucagon, the cAMP-lowering effect of 991 was completely abolished (Fig. 4b). Other compounds used were the PDE3-specific inhibitor cilostamide and the pan-PDE inhibitor IBMX, which, along with rolipram, were added to the assays for measuring PDE activity in hepatocyte extracts. PDE activity in control extracts was inhibited by about 70% by rolipram and by over 90% by IBMX, but was not significantly decreased by cilostamide (Fig. 4c), confirming that PDE4 is mainly responsible for cAMP removal in primary mouse hepatocytes. Moreover, PDE activation by 991 was not seen in the presence of rolipram (or IBMX), but was still present when assays were performed in the presence of cilostamide, suggesting that PDE4 was activated in response to 991 treatment. PDE assays routinely contained 1 μM cAMP, but the extent of PDE activation in hepatocytes incubated with 991 was the same when extracts were assayed with a saturating concentration of cAMP (50 μM), indicating an effect of 991 treatment on *V*_max_ rather than *K*_M_ (data not shown). Moreover, analysis of the effects of *in vitro* phosphorylation of purified recombinant PDE4B by AMPK on the kinetic properties of the enzyme revealed that *V*_max_ increased approximately twofold with no effect on *K*_M_ (see below).

### AMPK phosphorylates and activates mouse PDE4B *in vitro*

**Figure 5:**
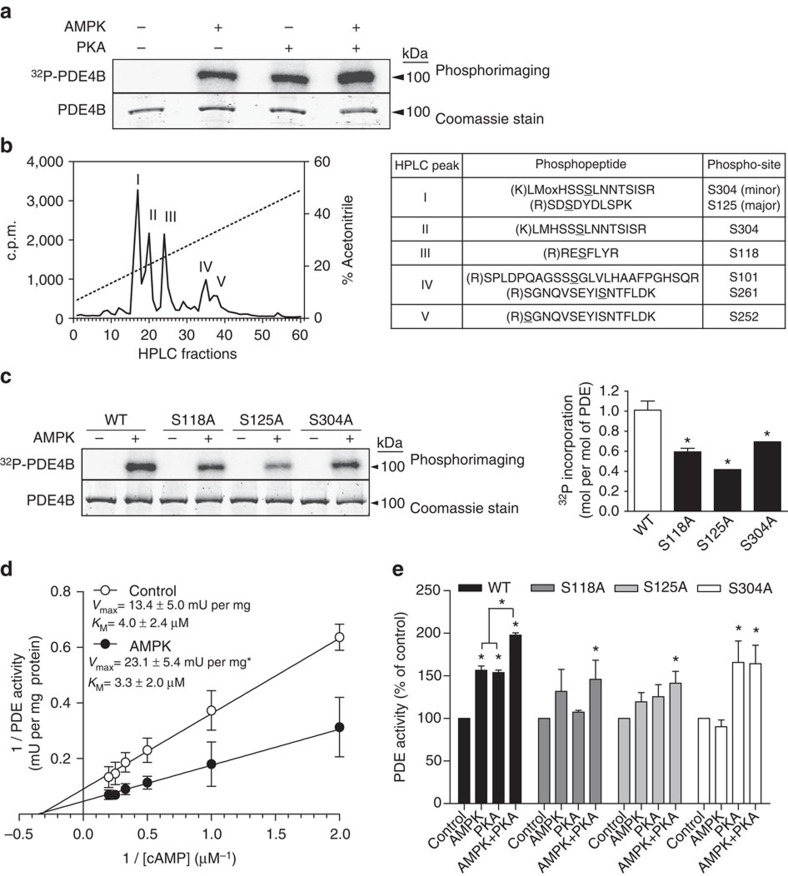
Phosphorylation-induced activation of mouse liver PDE4B. PDE4B was cloned from mouse hepatocyte cDNA. The recombinant protein was overexpressed *in E. coli* and purified. PDE protein was phosphorylated for 1 h with purified recombinant activated AMPK and/or purified PKA catalytic subunits and [γ-^32^P] ATP, and analysed by SDS–PAGE followed by Coomassie blue staining and phosphorimaging for quantification (**a**,**c**). In **b**, PDE was phosphorylated for 1 h with recombinant activated AMPK and [γ-^32^P]. Phosphorylation sites were identified by LC–MS/MS after trypsin digestion and radioactive peak separation by high-performance liquid chromatography (HPLC). The phosphorylation sites that were identified are underlined in the right hand panel. In **d** and **e**, recombinant PDE was phosphorylated as above but with non-radioactive ATP for PDE assay as indicated. In **d**, separate determinations of *V*_max_ and *K*_M_ were made by linear regression of double reciprocal (Lineweaver Burk) plots. In **e**, the basal PDE activities of the wild-type (WT), S118A, S125A and S304A mutant proteins were 1.97±0.25, 0.14±0.01, 1.59±0.15 and 0.32±0.09 mU per mg of protein, respectively. Values are means±s.e.m. for *n*=3 (**c**–**e**) separate experiments. Statistical analysis was by a paired Student's *t*-test. *Indicates a significant difference (*P*<0.05) compared with control incubations or between the indicated conditions.

To test whether PDE4B could be a direct AMPK substrate, we cloned the 721-amino-acid-containing PDE4B isoenzyme (GenBank: AF326555.1) from primary mouse hepatocyte total cDNA. The recombinant protein was expressed in *Escherichia coli* as a glutathione S-transferase (GST) fusion protein and purified before removing the tag by specific proteolytic cleavage. Both bacterially expressed recombinant activated AMPK and purified PKA catalytic subunits phosphorylated PDE4B in the presence of [γ-^32^P] ATP *in vitro* (Fig. 5a), and using AMPK, a stoichiometry of ∼1 mol of phosphate incorporated per mol of PDE protein was reached (Fig. 5c). With both AMPK and PKA, phosphorylation of PDE4B in the presence of [γ-^32^P] ATP was additive, suggesting the presence of distinct phosphorylation sites. After maximal phosphorylation by AMPK and [γ-^32^P] ATP, followed by trypsin digestion and peptide separation by HPLC, three AMPK phosphorylation sites were identified by liquid chromatography-coupled tandem mass spectrometry (LC–MS/MS) in the major radiolabelled peaks as Ser118, Ser125 and Ser304 (Fig. 5b). Ser118 is located in the upstream conserved regulatory region 1 of PDE4B, and was also phosphorylated by PKA, in agreement with previous reports of phosphorylation at this site leading to activation of long PDE4 isoforms^32^,^32^. Ser125 is also situated in upstream conserved regulatory region 1, while Ser304 corresponds to Ser245 located in the catalytic domain of PDE4D9 (ref. 34). The sequences surrounding Ser118, Ser125 and Ser304 are well conserved in vertebrate PDE4 orthologues (Supplementary Fig. 3A) and in the different mouse PDE4 isoforms (Supplementary Fig. 3B). When Ala residues were introduced by site-directed mutagenesis to replace each Ser, the stoichiometry of phosphorylation by AMPK decreased by 40–60% for the purified mutant recombinant proteins compared with wild-type PDE4B (Fig. 5c and Supplementary Fig. 4). *In vitro* phosphorylation of wild-type PDE4B by AMPK increased the *V*_max_ almost twofold without significantly affecting the *K*_M_ for cAMP, which was 3–4 μM (Fig. 5d). When recombinant PDE4B was incubated with ATP and AMPK or PKA, or the two protein kinases in combination, phosphorylation by AMPK or PKA led to a ∼50% increase in wild-type PDE4B activity, and an additive effect (twofold activation) was observed when both protein kinases were included together (Fig. 5e). AMPK-induced PDE4B activation was completely abolished in the S304A mutant, suggesting that phosphorylation at this site is crucial for the increase in *V*_max_. PKA-induced PDE4B activation was maintained in the S304A mutant, but was abolished in the S118A mutant. Moreover, the additivity of PDE4B activation by PKA and AMPK in combination was lost in the S304A mutant. Interestingly, mutation of S118 or S304 to Ala substantially decreased basal PDE activity of recombinant PDE4B compared with the wild-type protein (Fig. 5e and legend), suggesting that Ser118 and Ser304 could be important for catalysis or play structural roles, as well as being important for phosphorylation-induced PDE activation. Taken together, the findings suggest that Ser304 of mouse PDE4B is probably the major activating phosphorylation site for AMPK *in vitro*.

### PDE4B is phosphorylated by AMPK in intact hepatocytes

**Figure 6:**
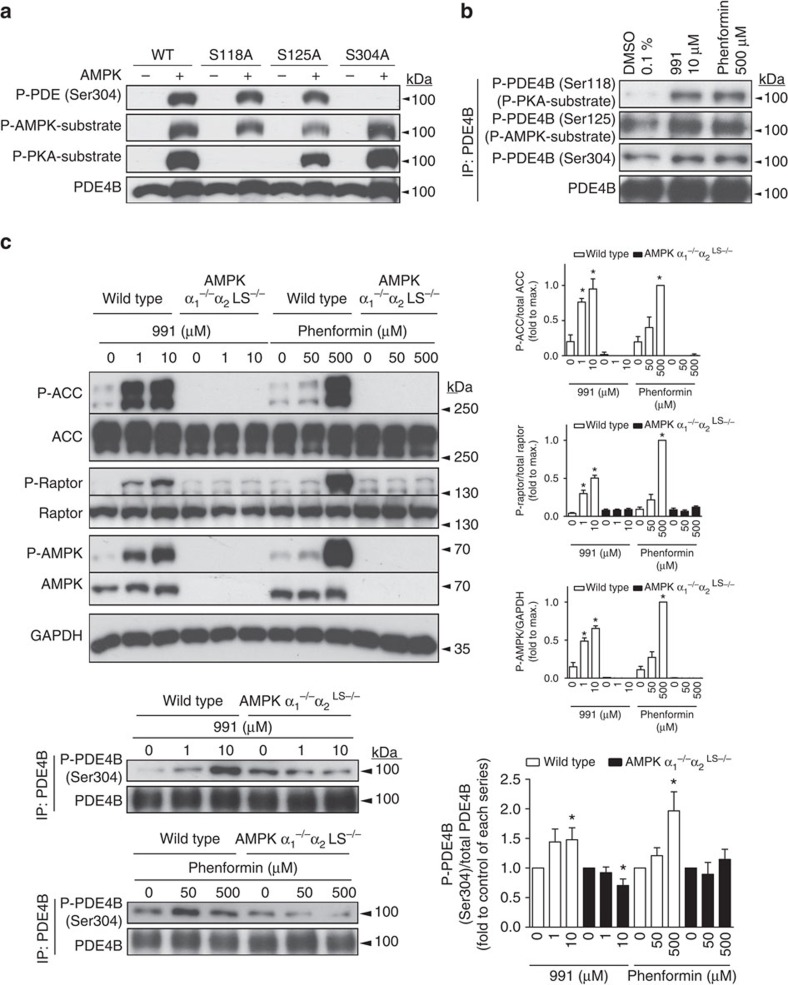
AMPK activation leads to PDE4B phosphorylation in intact hepatocytes. In **a**, wild-type (WT) or mutant recombinant mouse liver PDE4B was incubated for 1 h with non-radioactive ATP in the presence (+) or absence (−) of recombinant activated AMPK. Proteins (0.1 μg) were seperated by SDS–PAGE for immunoblotting with the indicated antibodies. In **b** and **c**, mouse hepatocytes from either WT (**b**) or both WT and AMPK α_1_^−/−^α_2_^LS−/−^ mice (**c**) were serum-starved overnight and incubated for 1 h with the indicated concentrations of 991 or phenformin. The cells were collected and lysed for immunoblotting with the indicated antibodies, except for PDE4B, which was immunoprecipitated as described in the Methods section, before immunoblotting. In **c**, phosphorylation levels of AMPK and its targets ACC, Raptor and PDE4B were quantified by densitometry and expressed relative to the corresponding total protein levels or GAPDH before normalization as indicated. Representative immunoblots are shown and for blot quantification in **c**, the values are means±s.e.m. for *n*=3 (p-ACC, p-Raptor and p-AMPK) or *n*=4 (p-PDE4B) separate experiments. Statistical analysis was by a paired Student's *t*-test. *Indicates a significant difference (*P*<0.05).

Finally we looked whether PDE4B could be phosphorylated by AMPK in intact cells. PDE4B was immunoprecipitated from primary mouse hepatocytes incubated with either 991 or phenformin, and extracts were subjected to immunoblotting with phospho-specific antibodies. Commercial antibodies against phosphorylation site peptide motifs for either PKA (to recognize PDE4B phosphorylated at Ser118) or AMPK (to recognize PDE4B phosphorylated at Ser125), as well as an anti-phosphopeptide antibody raised against the sequence surrounding Ser304 that we generated ourselves were used. The specificities of the anti-phospho antibodies were first verified on recombinant PDE4B phosphorylated by AMPK *in vitro* (Fig. 6a). Following immunoprecipitation of endogenous PDE4B from intact hepatocytes incubated with 991 or phenformin and immunoblotting, phosphorylation increased at the three main sites we identified (Fig. 6b), although some basal phosphorylation was seen in control-incubated hepatocytes. In hepatocytes from wild-type mice incubated with increasing concentrations of 991 or phenformin up to maximal doses, phosphorylation of AMPK, ACC and Raptor was increased, and this increase was completely abrogated or reduced in hepatocytes from AMPK α_1_^−/−^α_2_^LS−/−^ mice (Fig. 6c). Again, although some basal PDE4B phosphorylation at the activating site Ser304 was seen in untreated hepatocytes, incubation of hepatocytes with the highest doses of 991 and phenformin led to significant increases in PDE4B Ser304 phosphorylation, which were lost in hepatocytes from AMPK α_1_^−/−^α_2_^LS−/−^ mice (Fig. 6c). Basal PDE4B Ser304 phosphorylation, which was also apparent in hepatocytes lacking AMPK, suggests that kinase(s) other than AMPK could phosphorylate PDE4B. It is noteworthy that members of the AMPK-related salt-inducible kinase (SIK) family were shown to be involved in the regulation of hepatic gluconeogenesis^35^,^36^, and SIK1 was recently reported to activate mouse PDE4D in pancreatic β-cells via phosphorylation of Ser136 (ref. 37), the residue corresponding to Ser125 of PDE4B identified here.

## Discussion

**Figure 7:**
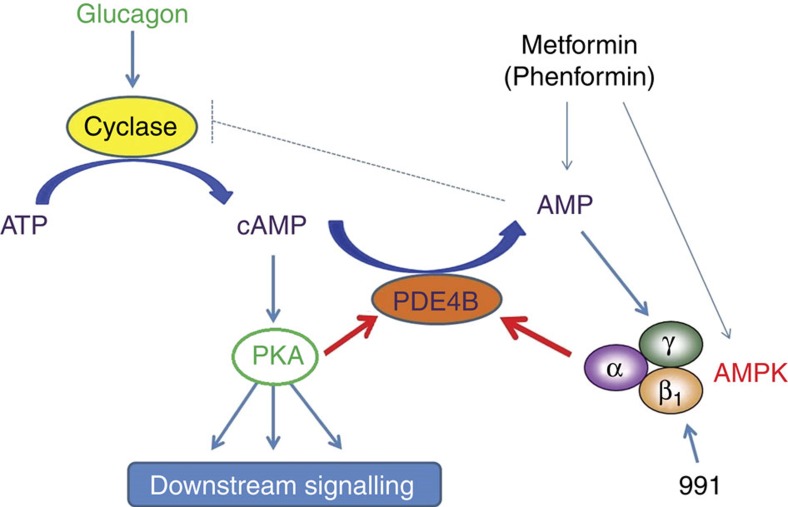
How biguanides and compound 991 antagonize glucagon signalling. Unlike biguanides, treatment with 991 activates AMPK without increasing cellular AMP levels. Both biguanides and 991 activate the major PDE 4B isoenzyme in hepatocytes in an AMPK-dependent manner. Metformin and phenformin activate hepatic AMPK either directly or via a rise in AMP, which could compete with ATP to inhibit adenylate cyclase. Phosphorylation-induced activation of PDE4B by AMPK reduces glucagon-stimulated cAMP accumulation. As a consequence, PKA activation by glucagon and downstream signalling are decreased in hepatocytes incubated with 991, the effect being AMPK-dependent.

In the present study, we propose that hepatic AMPK activation leads to phosphorylation-induced PDE4B activation, thereby antagonizing the glucagon-stimulated rise in cAMP and PKA signalling (summarized in Fig. 7). This proposal is based on the following evidence: (1) treatment of hepatocytes with the small-molecule AMPK activator 991 decreased glucagon-induced increases in cAMP (Fig. 1a,e) by accelerating cAMP removal (Fig. 2c) without affecting intracellular adenine nucleotide levels (Fig. 2a); (2) compound 991 treatment led to a stable increase in PDE activity (Fig. 2d,e), and inclusion of the PDE inhibitors IBMX and rolipram in hepatocyte incubations abolished the effect of 991 to reduce increases in cAMP levels in response to glucagon (Figs 2f and 4b); (3) AMPK phosphorylated purified PDE4B *in vitro* (Fig. 5a–c), resulting in an increase in *V*_max_ (Fig. 5d), and mutation of Ser304 to Ala abolished phosphorylation-induced PDE activation by AMPK (Fig. 5e); and (4) 991 treatment led to increased PDE4B Ser304 phosphorylation in intact hepatocytes (Fig. 6b). Importantly, in hepatocytes from mice bearing a liver-specific deletion of the two AMPK catalytic subunits, the effect of 991 treatment to decrease cAMP levels and subsequent PKA activation in response to glucagon was lost (Fig. 3b,c) along with PDE activation (Fig. 3d) and increased PDE4B Ser304 phosphorylation (Fig. 6c).

Interestingly, the AMPK phosphorylation sites in PDE4B we identified correspond to recently described phosphorylation sites in PDE4D9, and Ser245 of human PDE4D9 (Ser304 in PDE4B3) was proposed as an activating site phosphorylated by an unknown ‘switch kinase' activated by H_2_O_2_ (ref. 34). Also, in a phosphoproteomics study^38^, PDE4C from hepatocytes was found to be an AMPK substrate and the phosphorylation site identified corresponds to Ser125 of PDE4B, present in all long PDE4 isoforms (Supplementary Fig. 3B). Moreover, phosphorylation sites Ser118, Ser125 and Ser304 are well conserved in vertebrate PDE4B orthologues (Supplementary Fig. 3A). Phosphorylation at Ser304 is probably responsible for the increase in *V*_max_ of PDE4B, since its mutation to Ala abolished AMPK-induced PDE activation (Fig. 5d). However, as reported for PDE4D9 (ref. 34), multi-site phosphorylation of PDE4B might be necessary for full PDE activation, and from our data we cannot rule out the involvement of Ser118 and Ser125 phosphorylation in overall AMPK-induced PDE4B activation. Phosphorylation-induced PDE4 activation by AMPK might be of broad physiological relevance, since PDE4 (especially isoforms B and D) has a wide tissue distribution^39^.

The concentrations of cAMP reached in our study in cultured mouse hepatocytes incubated with glucagon (up to 20 pmol per mg of protein in the absence of PDE inhibitors, Figs 1e, 2c and 3b) fit well with values reported in the literature^40^,^41^,^42^,^43^. These values were obtained using a commercial ELISA-based assay and were much lower than those measured by radioimmunoassay (∼150 pmol per mg of protein, see for example Fig. 1a) or by HPLC (∼100 pmol per mg of protein, data not shown) when the three methods were compared on the same sample. Such differences are likely to be due to differences in sample quenching. For radioimmunoassay, hepatocyte incubations were immediately stopped with HCl. For HPLC, the cells were immediately extracted with perchloric acid, whereas for measurements by ELISA, the stopping mixture used for rapid lysis of hepatocytes was a non-deproteinizing buffer containing detergent and EDTA. Radioimmunoassay and HPLC would thus give measurements of total cAMP levels, whereas ELISA would provide measurements of "free" concentrations. However, effects of 991 to decrease glucagon-stimulated increases in cAMP were observed irrespective of the protocol used for cAMP measurement. Increased PDE activity due to AMPK activation would correspond to 5-10 pmol per min per mg of protein (Figs 2d and 3d), which would be sufficient to account for the degradation of cAMP as measured by ELISA in 991-stimulated hepatocytes incubated for 15 min, with glucagon (Fig. 2c).

Whole-body deletions of PDE4B have been made in mice, associated with inhibition of the lipopolysaccharide-stimulated immune response of peripheral leukocytes^44^, increased Ca^2+^ transients and contractility of cardiac myocytes^45^ and reduced adiposity and high-fat-diet-induced adipose inflammation^46^. PDE4B^−/−^ mice were reported to be normal in terms of body weight and growth rate, showing no obvious abnormalites^44^. Also during starvation, serum glucose/insulin levels as well as glucose/insulin tolerance were not altered in these mice^46^. However, effects on liver metabolism were apparently not investigated in these studies.

The decrease in glucagon-stimulated cAMP accumulation seen in hepatocytes incubated with phenformin was proposed to be due to direct inhibition of adenylate cyclase by AMP, with a half-maximal effect at about 300 μM (ref. 21). In hepatocytes incubated with 500 μM phenformin, AMP concentrations rose to over 1 mM (ref. 21), a value comparable to that of ∼2 nmol per mg of protein seen here (Fig. 2a), which would correspond to ∼0.5 mM. However, free cytosolic AMP concentrations are much less than total cellular concentrations because AMP is bound by abundant proteins, such as glycogen phosphorylase, adenylate kinase, 6-phosphofructo-1-kinase and fructose-1,6-bisphosphatase^47^. The free AMP concentration in livers of wild-type mice was calculated to be 3.9 nmol per g of wet weight^48^, which would correspond to 7 μM AMP based on the intracellular water space of perfused liver^49^. Therefore, even under conditions of ATP depletion induced by phenformin, it is unlikely that free AMP concentrations would rise to levels high enough to inhibit adenylate cyclase. Although we cannot rule out phenformin acting via direct inhibition of adenylate cyclase by AMP, PDE activation could also explain the reduction in glucagon-stimulated cAMP levels by biguanides previously reported^21^ (see scheme, Fig. 7). Indeed, we show that submaximal concentrations of 991, phenformin and metformin activated PDE and the increase in PDE activity correlated with AMPK activation due to compound treatment (Supplementary Fig. 1).

In summary, by analogy with insulin signalling^28^, activation of AMPK inhibits glucagon-stimulated cAMP accumulation by activating a PDE. Interestingly, effects of metformin to lower blood glucose levels are only seen in diabetic and not in normal subjects^50^, consistent with increased circulating glucagon concentrations in diabetic individuals, who presumably would have elevated cAMP levels in liver. Our data suggest that AMPK activation in the liver could be beneficial by opposing short-term glucagon action via PDE activation to reduce cAMP as a new therapeutic strategy for the treatment of metabolic diseases associated with dysregulated cAMP/PKA signalling, such as type 2 diabetes. It is also noteworthy that cAMP signalling is important for the growth of certain cancers and that suppression of negative feedback mechanisms occurs during tumorigenesis^51^.

## Methods

### Reagents and materials

Compound 991 (previously referred to as ex229 (ref. 25) from patent application WO2010036613, Merck Sharp & Dohme Corp., Metabasis Therapeutics, Inc. Novel cyclic benzimidazole derivatives useful anti-diabetic agents, 2010) was kindly provided by AstraZeneca Mölndal, SE. Glucagon (reconstituted GlucaGen) was from Novo Nordisk, phenformin and all other reagents were from Sigma Aldrich. All cell culture reagents were from Life Technologies. Oligonucleotides were from Eurogentec. [γ-^32^P] ATP and [5′, 8′-^3^H] cAMP were from Perkin Elmer. Anti-total ACC (Merck Millipore, Catalogue No. 04-322), anti-P-Ser79-ACC (Merck-Millipore, Catalogue No. 07-303), anti-glyceraldehyde-3-phosphate dehydrogenase (GAPDH; Merck-Millipore, Catalogue No. MAB374), anti-total GP (Sigma, Catalogue No. HPA000962), anti-total AMPK β1(R&D Systems, Catalogue No. AF2854) and anti-total AMPK β2 (R&D Systems, Catalogue No. MAB3808), anti-PThr172-AMPK (T172) (Cell Signaling Technologies, Catalogue No. 2535, anti-P-AMPK-substrate (Cell Signaling Technologies, Catalogue No. 5759), anti-P-PKA-substrate (Cell Signaling Technologies, Catalogue No. 9624), anti-total Raptor (Cell Signaling Technologies, Catalogue No. 2280) and anti-P-Ser792-Raptor (Cell Signaling Technologies, Catalogue No. 2083), anti-total CREB (Cell Signaling Technologies, Catalogue No. 9197), anti-phospho-Ser133-CREB (Cell Signaling Technologies, Catalogue No. 9198) and anti-total PDE4B (Origene, Catalogue No. TA503471) antibodies were from the sources cited. Anti-P-Ser14-GP and anti-total AMPK (α1 and α2) antibodies were kindly provided by Grahame Hardie (University of Dundee, UK). A peptide surrounding Ser304 of mouse PDE4B (CKLMHSSSLNNTSI) was synthesized with or without Ser304 phosphorylated and with a Cys (N-term) for coupling to keyhole limpet haemocyanin (KLH) or bovine serum albumin (BSA) (Imject maleimide-activated KLH/BSA kit, Thermo Fisher Scientific). The KLH-coupled phosphopeptide was injected in rabbits (Thermo Fisher Scientific), and the serum was affinity purified on both BSA-coupled phosphopeptide and non-phosphopeptide linked to CH-activated Sepharose 4B (GE Healthcare). Catalytic subunits of PKA were purified from bovine heart as described previously^52^. Recombinant bacterially expressed AMPK (α_1_β_1_γ_1_) was activated with recombinant bacterially expressed LKB1-MO25-STRAD complex^52^, both kindly provided by Dietbert Neumann (Maastricht University, Maastricht, NL). pGEX6p1 vector was generously donated by Christopher Proud (South Australian Health & Medical Research Institute, University of Adelaide, Australia). Synthetic peptides were provided by Vincent Stroobant (LICR, Brussels, BE). Oligonucleotide primers were synthetized by Integrated DNA Technologies.

### Animals

Animal experiments were approved by the Université catholique de Louvain Brussels local ethics committee and conducted within the guidelines of the European Convention for the Protection of Vertebrate Animals used for Experimental and Other Scientific Purposes. Male C57BL/6 wild-type mice (3–4 months old) were obtained from the local animal facility and maintained on a 12 h light/12 h dark cycle with free access to food and water. Liver-specific AMPK α_1_/α_2_ knockout mice were generated^53^, and experiments on AMPK α_1_/α_2_ double knockout and control mice were performed under the approval of the ethics committee from University Paris Descartes (No. CEEA34.BV.157.12) and the French authorization to experiment on vertebrates (No.75-886) in accordance with European guidelines. The AMPKβ1 knockout-mouse strain was generated by the trans-NIH Knock-Out Mouse Project (KOMP) and obtained from the KOMP Repository (www.komp.org). These mice were used in accordance with the United Kingdom Animals (Scientific Procedures) Act (1986).

### Primary mouse hepatocyte culture and incubation

Mice were anesthetized (pentobarbital injection, i.p.) and livers were washed (with 50 ml of Krebs-HEPES, pH 7.65, supplemented with 0.5 mM EDTA) and digested (with 50 ml of Krebs-HEPES, pH 7.65, containing 25 mg of collagenase from *Clostridium hystolyticum* and 0.5 mM CaCl_2_) by perfusion through the inferior vena cava at a rate of 5 ml min^−1^ as described^18^. The liver was removed and hepatocytes were extracted in attachment medium (DMEM supplemented with 1 g l^−1^ glucose, 4 mM glutamine, 1 mM pyruvate, penicillin/streptomycin, 10% (v/v) FBS, 10 nM insulin, 200 nM triiodothyronine (T3) and 500 nM dexamethasone). After filtering through a 100-μm mesh cell strainer (BD Falcon), cells were pelleted (50*g* × 2 min) and resuspended in attachment medium for counting and seeding. Typically, cells were distributed in six-well plates, 2 ml per well containing 2.5 × 10^5^ cells. After attachment for 4 h, the cells were washed in PBS and incubated for 20 h in overnight medium (DMEM containing 1 g l^−1^ glucose, 4 mM glutamine, 1 mM pyruvate, penicillin/streptomycin and 100 nM dexamethasone). Before treatment, the medium was replaced with fresh overnight medium. Unless otherwise stated, the cells were incubated first with 10 μM 991 (or 0.1% (v/v) DMSO, vehicle controls) for 20 min before treatment with the indicated concentrations of glucagon (or PBS, vehicle controls) for 15 min.

### Cell lysis and immunoblotting

Following treatment in six-well plates, the medium was removed and the cells were washed in cold PBS before lysis in buffer containing 50 mM HEPES, pH 7.5, 1 mM EDTA, 1 mM EGTA, 0.5% (v/v) 2-mercaptoethanol, 50 mM NaF, 5 mM Na_4_P_2_O_7_, 5 mM sodium β-glycerophosphate, 1 mM Na_3_VO_3_, 1 mM dithiothreitol, 0.1% (w/v) Triton X-100 and Complete protease inhibitor cocktail (150 μl per well). Extracts were centrifuged (20,000*g* × 5 min at 4 °C) and protein concentrations were measured. For immunoprecipitation of PDE4B, 1 mg of extract protein was incubated for 2 h at 4 °C with 50 μl of protein G-Sepharose (GE Healthcare) previously coupled to 2 μg of anti-PDE4B antibody. For immunoblotting, 10 μg of sample protein or eluted immunoprecipitated proteins were loaded on 7.5% (w/v) polyacrylamide gels. Following SDS–PAGE, proteins were transferred to polyvinylidene difluoride membranes, which were then blocked in Tris-buffered saline (TBS) containing 0.1% (v/v) Tween and 5% (w/v) BSA. The membranes were incubated overnight at 4 °C with the indicated primary antibodies diluted in blocking buffer, then washed extensively before and after incubation for 1 h with horseradish peroxidase-conjugated secondary antibodies. Anti-total and anti-P-ACC, anti-total GP, anti-total AMPK (β1 and β2), anti-P-AMPK, anti-P-AMPK-substrate, anti-P-PKA-substrate, anti-total and anti-P-Raptor, anti-total and anti-P-CREB antibodies were used at a dilution of 1:1,000. Anti-GAPDH and anti-total PDE4B antibodies were used at dilutions of 1:30,000 and 1:500, respectively. Anti-P-PDE4B (S304), P-GP and anti-total AMPK (α1 and α2) antibodies were used at dilutions of 1:1,000, 1:1,000 and 1:10,000, respectively. For incubation of membranes with the anti-P-PDE4B (S304) antibody, the non-phosphopeptide used for antibody purification was included at a concentration of 10 μg ml^−1^. Immunodetection was by ECL Classico substrate (Merck Millipore). Immunoblots were quantified by densitometry using ImageJ software. Uncropped scans of the blots used to generate the figures are shown in Supplementary Fig. 5.

### cAMP measurement

cAMP was measured using the ELISA-based cAMP XP assay kit (Cell Signaling Technologies) according to the manufacturer's protocol. Following hepatocyte incubations in six-well plates, the cells were lysed in 100 μl of kit buffer (20 mM Tris-HCl, pH 7.5, 150 mM NaCl, 1 mM EDTA, 1 mM EGTA, 1% (w/v) Triton, 2.5 mM Na_4_P_2_O_7_, 1 mM sodium β-glycerophosphate, 1 mM Na_3_VO_4_, 1 μg ml^−1^ leupeptin and 1 mM phenylmethyl sulfonyl fluoride). The samples were diluted in lysis buffer in experiments with PDE inhibitors. After centrifugation (20,000*g* × 5 min at 4 °C), 50μl aliquots of supernatant were taken and added to 50 μl of horseradish peroxidase-cAMP kit solution (unknown composition) for cAMP measurement. Quantification was based on a linear concentration curve, established with external cAMP standards. Alternatively, 1 ml of 0.1 M HCl was added to each well for cell lysis, and cAMP was measured in the dried supernatant by radioimmunoassay using a home-made antibody after sample acetylation^54^.

### Enzyme assays

PKA activity in hepatocyte lysates was measured by phosphorylation of a substrate peptide derived from rat heart 6-phosphofructo-2-kinase (PFK-2, sequence PVRMRRNSFT) in the presence and absence of PKA inhibitor peptide (PKI, sequence TYADFIASGRTGRRNAIHD). Lysates prepared for immunoblotting (corresponding to 30 μg of protein) were assayed at 30 °C in 30 μl of phosphorylation buffer containing 10 mM MOPS, pH 7.0, 0.5 mM EDTA, 10 mM magnesium acetate, 5 mM dithiothreitol, 100 μM substrate peptide and 100 μM [γ-^32^P] ATP (specific radioactivity 1,000 c.p.m. pmol^−1^) with and without 10 μM PKI. After 5 min, aliquots (20 μl) were spotted on P81 phosphocellulose papers for the determination of ^32^P-incorporation. AMPK (α1 plus α2) activity immunoprecipitated from hepatocyte lysates was measured as described^55^,^56^. Purified PKA and recombinant activated AMPK were assayed in phosphorylation buffer by measuring ^32^P incorporation from 0.1 mM [γ-^32^P] ATP (specific radioactivity 1,000 c.p.m. pmol^−1^) into 200 μM AMARA peptide (AMARAASAAALRRR) for AMPK and 200 μM the peptide PVRMRRNSFT for PKA^52^. One unit of protein kinase activity corresponds to the amount that catalyses the formation of 1 nmol min^−1^ of product under the assay conditions.

GP activity was determined in the direction of glycogen synthesis as described^57^. Lysates prepared for immunoblotting (corresponding to 100 μg of protein) were assayed in 100 μl of reaction mixture containing 25 mM Tris-HCl, pH 6.1, 50 mM glucose 1-phosphate, 1% (w/v) purified oyster glycogen, 150 mM NaF and 0.5 mM caffeine for 20 min at 30 °C. Blanks for spontaneous glucose 1-phosphate hydrolysis contained lysis buffer. Reactions were stopped by adding 100 μl of ice-cold 1.2 M trichloroacetic acid, and the samples were centrifuged (20,000*g* × 5 min at 4 °C). Inorganic phosphate in the supernatant was measured spectrophotometrically at 650 nm 30 min after adding 20 mM 8-anilinonaphthalene-1-sulfonic acid and 2 mM ammonium molybdate in a final volume of 1 ml.

PDE in aliquots of lysates prepared for immunoblotting (containing about 50 μg of protein) or 0.5 μg of tag-removed recombinant PDE (see below) was assayed as described^58^ for 10 min at 30 °C. The reaction mixture contained 40 mM Tris-HCl, pH 8.1, 0.5% (v/v) 2-mercaptoethanol, 5 mM MgCl_2_, 0.1% (w/v) BSA and 1 μM [5′, 8′-^3^H] cAMP (specific radioactivity 400 d.p.m. pmol^−1^) in a final volume of 500 μl. For determination of the effect of phosphorylation by AMPK on the kinetic properties of recombinant PDE, cAMP concentrations were varied between 0.5 and 5 μM with a fixed amount of [5′, 8′-^3^H] cAMP (1,000 d.p.m. μl^−1^). Blanks for spontaneous cAMP hydrolysis contained the corresponding buffer. Reactions were stopped by freezing in liquid nitrogen followed by thawing for 1 min in boiling water. The reaction product, radioactive AMP, was further hydrolysed with 0.2 U of snake venom 5′-nucleotidase for 10 min at 30 °C. Residual cAMP and AMP were retained on 1 ml Dowex-formate columns (0.5 × 10 cm, Biorad), and radioactive adenosine in the flow-through was collected after further elution with 2 ml of CH_3_OH for liquid scintillation counting. One unit of GP or PDE activity corresponds to the amount that catalyses the formation of 1 μmol min^−1^ of product under the assay conditions.

### Nucleotide measurements

Cells in six-well plates were lysed with ice-cold 50 mM perchloric acid (500 μl per well). Following centrifugation (20,000*g* × 10 min at 4 °C) the supernatants were neutralized with ∼60 μl of 1.1 M NH_4_H_2_PO_4_. The samples were then vacuum-dried and resuspended in 20 μl of water for HPLC separation and quantification of purine nucleotides as described^59^.

### Real-time PCR

Total mRNA was extracted from untreated primary mouse hepatocytes in six-well plates using 1 ml per well of Trizol (Life Technologies) reagent, according to the manufacturer's instructions. RNA was quantified by Nanodrop, and 500 ng was used for cDNA synthesis using the M-MLV RT kit (Life Technologies) according to the manufacturer's protocol. Reverse transcription products were diluted 1:3 in nuclease-free water, and 1 μl was used for qPCR with the Kapa Sybr Fast qPCR kit (Kapa Biosystems) in combination with the CFX96 Real Time PCR thermocycler (Biorad). The programme included 40 cycles at 95 °C for 10 s, 66 °C for 10 s, 72 °C for 30 s and a final melting curve. PCR reactions were carried out in a final volume of 10 μl with 1 μM of each of the primers against the different mouse PDE isoforms as described^60^.

### Expression and purification of mouse recombinant PDE4B

Total mRNA was extracted and retro-transcribed as mentioned above. cDNA product (1 μl) was used as a template for PCR, using Q5 High Fidelity DNA polymerase (New England Biolabs) according to the manufacturer's protocol and with the following oligonucleotide primers: (F) 5′-ggaaggatccATGACAGCAAAAAATTCTCC-3′ and (R) 5′-gctactcgagTTATGTGTCGATCGGAGACT-3′. PCR products were gel-purified with Wizard SV gel and PCR clean-up system (Promega), and verified by sequencing to confirm a 721-amino-acid protein with 95% identity to human PDE4B3. The sequence was cloned into the pGEX6p1 vector using the BamHI and XhoI restriction sites present in the primers. Site-directed mutagenesis was carried out using the Herculase II Fusion DNA polymerase (Stratagen), with the QuikChange (Agilent) protocol. GST-tagged PDE4B was expressed overnight at 18 °C in BL21 *E. coli* cells induced with 0.5 mM isopropylthiogalactoside. Bacteria were then collected by centrifugation (5,000*g* × 10 min at 4 °C), resuspended in 1/10 of the culture volume of ice-cold lysis buffer (50 mM Tris-HCl, pH 7.5, 150 mM NaCl, 0.1% (v/v) 2-mercaptoethanol, 0.01% (w/v) Brij 35, 0.5 mM phenylmethyl sulfonyl fluoride, 0.5 mM benzamidine Cl, 1 μg ml^−1^ leupeptin and 1 μg ml^−1^ aprotinin) and homogenized using a French press device. The lysate was cleared by centrifugation (17,000*g* × 20 min at 4 °C) and the supernatant was passed through a 45-μm mesh filter (Millex-HA, Merck-Millipore) before loading onto a GSH-Sepharose column (1 × 20 cm, GE Healthcare). After extensive washing, the column was eluted with a 0–10 mM gradient of GSH. Fractions were subjected to SDS–PAGE followed by Coomassie Blue staining. Fractions containing GST-PDE4B protein bands were pooled and concentrated using a 100-kD ultrafiltration unit (Amicon) while changing the buffer to enzyme storage buffer (50 mM Tris-HCl, pH 7.5, 150 mM NaCl, 0.1% (v/v) 2-mercaptoethanol and 10% (v/v) glycerol). The GST tag was removed overnight at 4°C by incubation of 500 μg of fusion protein with 10U of HRV 3C protease (Sino Biological) according to the manufacturer's protocol.

### *In vitro* phosphorylation

Recombinant PDE4B (1 μg) was phosphorylated with 0.4 U of purified activated recombinant bacterially expressed AMPK or purified PKA catalytic subunits in 20 μl of kinase assay buffer (see PKA assay) containing 200 μM AMP (for AMPK only) and 100 μM [γ-^32^P] ATP (specific radioactivity 1,000 c.p.m. pmol^−1^). For kinetic studies, incubations contained non-radioactive ATP at the same final concentration. After 1 h at 30 °C, the reaction was stopped on ice, and 10 μl of mixture was taken for further analysis. For kinetic studies, samples were diluted fivefold in enzyme storage buffer before PDE assay. For measurements of the stoichiometry of phosphorylation, proteins were separated by SDS–PAGE in 7.5% (w/v) polyacrylamide gels. Gels were stained using PageBlue (Thermo Fisher Scientific) for protein band quantification by Odyssey infrared imaging. Bands were then excised from the gels for measurement of ^32^P-incorporation by liquid scintillation as described previously^52^.

### Phosphorylation site identification

Recombinant PDE (3 μg) was phosphorylated as described above with 1 U of activated AMPK in a final volume of 60 μl for 1 h at 30 °C. The reaction was stopped on ice, 20 μg of BSA was added as carrier and proteins were precipitated with a final concentration of 10% (w/v) trichloroacetic acid for 45 min on ice. Precipitated proteins were collected by centrifugation (20,000*g* × 10 min at 4 °C), washed in acetone, vacuum-dried and resuspended in 50 μl of 50 mM NH_4_HCO_3_, pH 8.0, for overnight digestion at 30 °C with trypsin. Peptides were separated by reverse-phase narrow-bore HPLC and radioactive peaks were analysed by LC–MS/MS as described^61^. Multi-stage activation was enabled for phosphate neutral loss of 98, 49 or 32.66 with respect to the precursor *m*/*z*. Peak lists were generated and searched using SequestHT and PhosphoRS 3.1 against a home-made protein database containing the human PDE sequences obtained from Uniprot and the different mutants used in this study. Phosphorylation site identification was performed as described^61^ and validated manually.

### Statistical analysis

The results are expressed as means±s.e.m. of at least three independent experiments. Unless otherwise stated, all two-group comparisons were tested for statistically significant differences using a paired two-sided Student's *t*-test, and *P*<0.05 was considered significant.

## Additional information

**How to cite this article:** Johanns, M. *et al.* AMPK antagonizes hepatic glucagon-stimulated cyclic AMP signalling via phosphorylation-induced activation of cyclic nucleotide phosphodiesterase 4B. *Nat. Commun.* 7:10856 doi: 10.1038/ncomms10856 (2016).

## Supplementary Material

Supplementary Information Supplementary Figures 1-5
