# Supplementary material for: AMPK antagonizes hepatic glucagon-stimulated cyclic AMP signalling via phosphorylation-induced activation of cyclic nucleotide phosphodiesterase 4B
Source: Nat Commun. 2016 Mar 8;7:10856. doi: 10.1038/ncomms10856 (PMC4786776; doi:10.1038/ncomms10856)

Supplementary Fig. 1: Submaximal doses of biguanides activate PDE in hepatocytes. Mouse hepatocytes were incubated with increasing concentrations of phenformin for 2 h or metformin for 24 h. The cells were harvested and lysed for measurement of total PDE activity in cell extracts and AMPK phosphorylation was quantified by immunoblotting (A). In (B), phosphorylated and total ACC and AMPK levels were measured by immunoblotting as indicated with GAPDH as loading control. The data are means ± S.E.M. of 3 separate experiments and in (B) representative immunoblots are shown. Statistical analysis was by a paired Student's *t*-test and * indicates a significant difference ($P<0.05$) compared to control incubations.

A

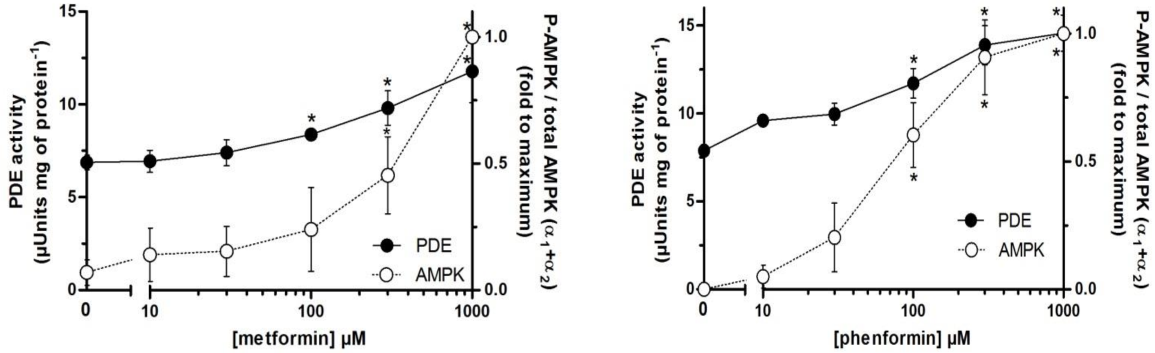

B

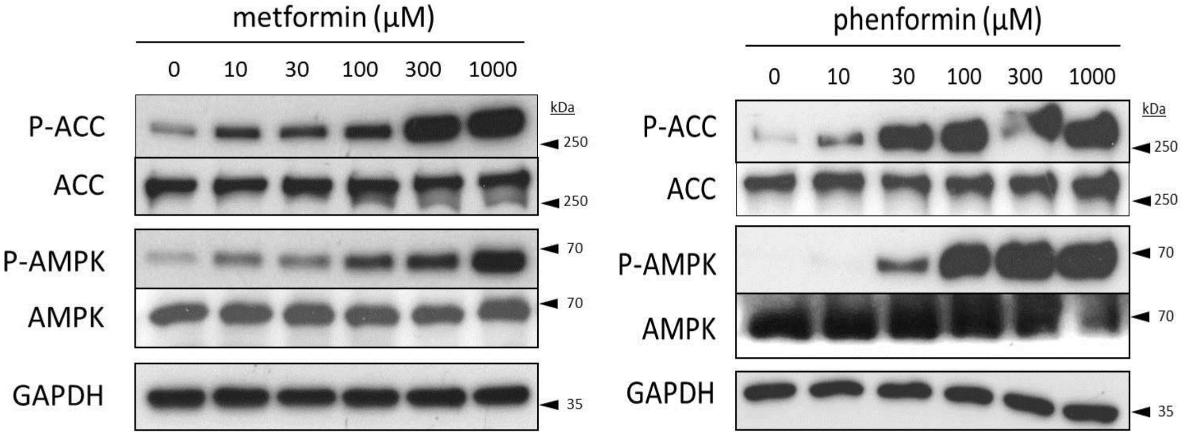

Supplementary Fig. 2: The decrease in glucagon-induced cAMP by 991 is abolished in hepatocytes from AMPK $\beta_1^{-/-}$ mice. Primary hepatocytes from wild-type mice or mice bearing a full-body deletion of the AMPK beta 1 subunit (AMPK $\beta_1^{-/-}$) were treated with 10 µM 991 (or DMSO as vehicle) prior to stimulation with 10 nM glucagon (Glg) for 15 min. The cells were harvested and lysed for the measurement of intracellular cAMP concentrations (A), and for immunoblotting levels of phosphorylated and total ACC, AMPK and Raptor (B) with GAPDH as loading control. The data are means ± S.E.M. of 3 separate experiments and in (B) representative immunoblots are shown. Statistical analysis was by a paired Student's *t*-test and * indicates a significant difference (P<0.05) compared to control incubations with DMSO.

A

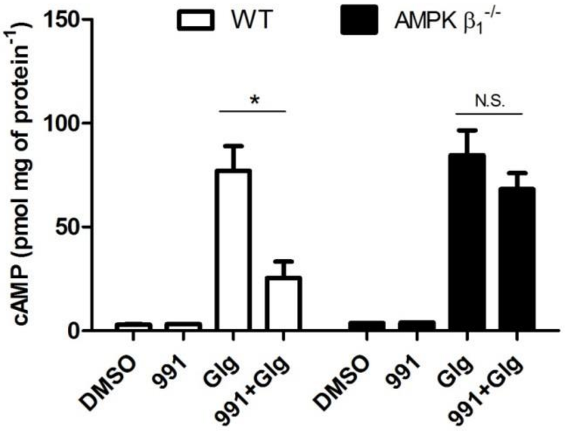

B

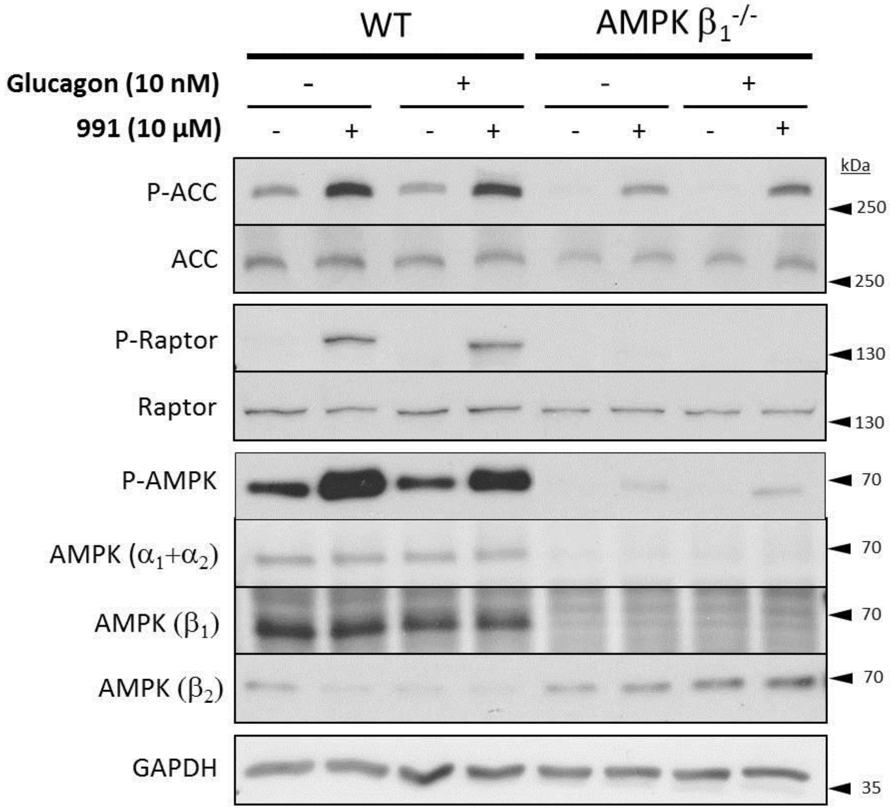

Supplementary Fig. 3: The three main AMPK phosphoryaltion sites in PDE4B are conserved and present in all PDE4 isoforms. Multiple sequence alignments were made using the Clustal W2 (EMBL-EBI) software with standard settings taking sequences from the Uniprot database for the PDE4B reference isoform from different species (A) and the mouse PDE4 A-D isoenzymes (B). The three AMPK phosphorylation sites identified (Ser118, Ser125 and Ser304) are shown in black boxes. The colors indicate different amino acid families (red: hydrophobic, green: polar uncharged, pink: positively charged, blue: negatively charged) and the symbols (*, :, .) beneath indicate the degree of conservation, in decreasing order.

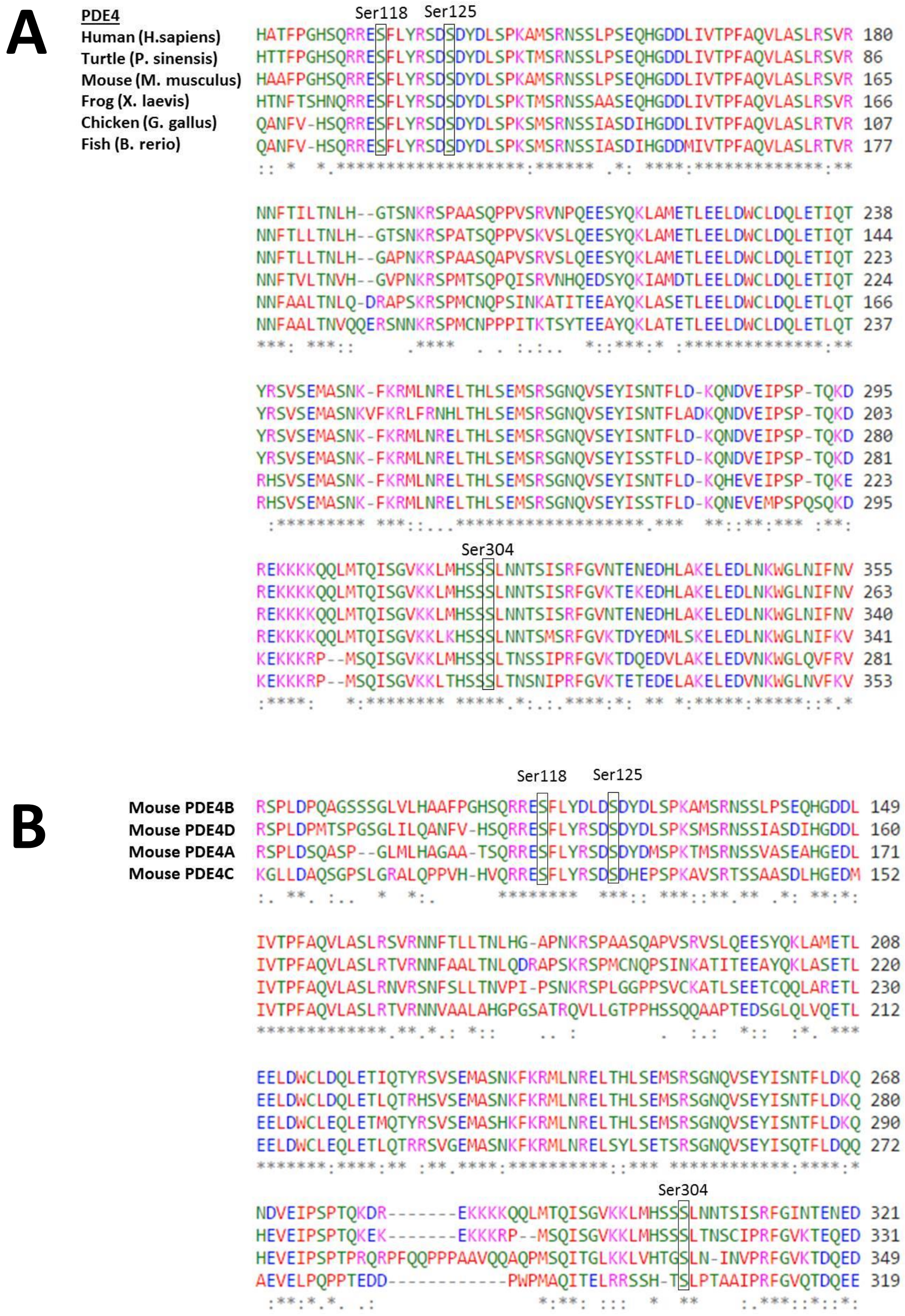

Supplementary Fig. 4: Radioactive HPLC profiles of PDE4B mutants phosphorylated by AMPK and digested with trypsin. Purified recombinant PDE4B mutant proteins were phosphorylated by activated AMPK and [γ-^{32}P] ATP as described in the legend to Fig. 5 of the main manuscript, where I, II and III indicate the three major peaks obtained for wild-type PDE4B. The arrows indicate the phosphorylation peak which disappears for each of the mutated Ser → Ala residues. The data are from one representative experiment.

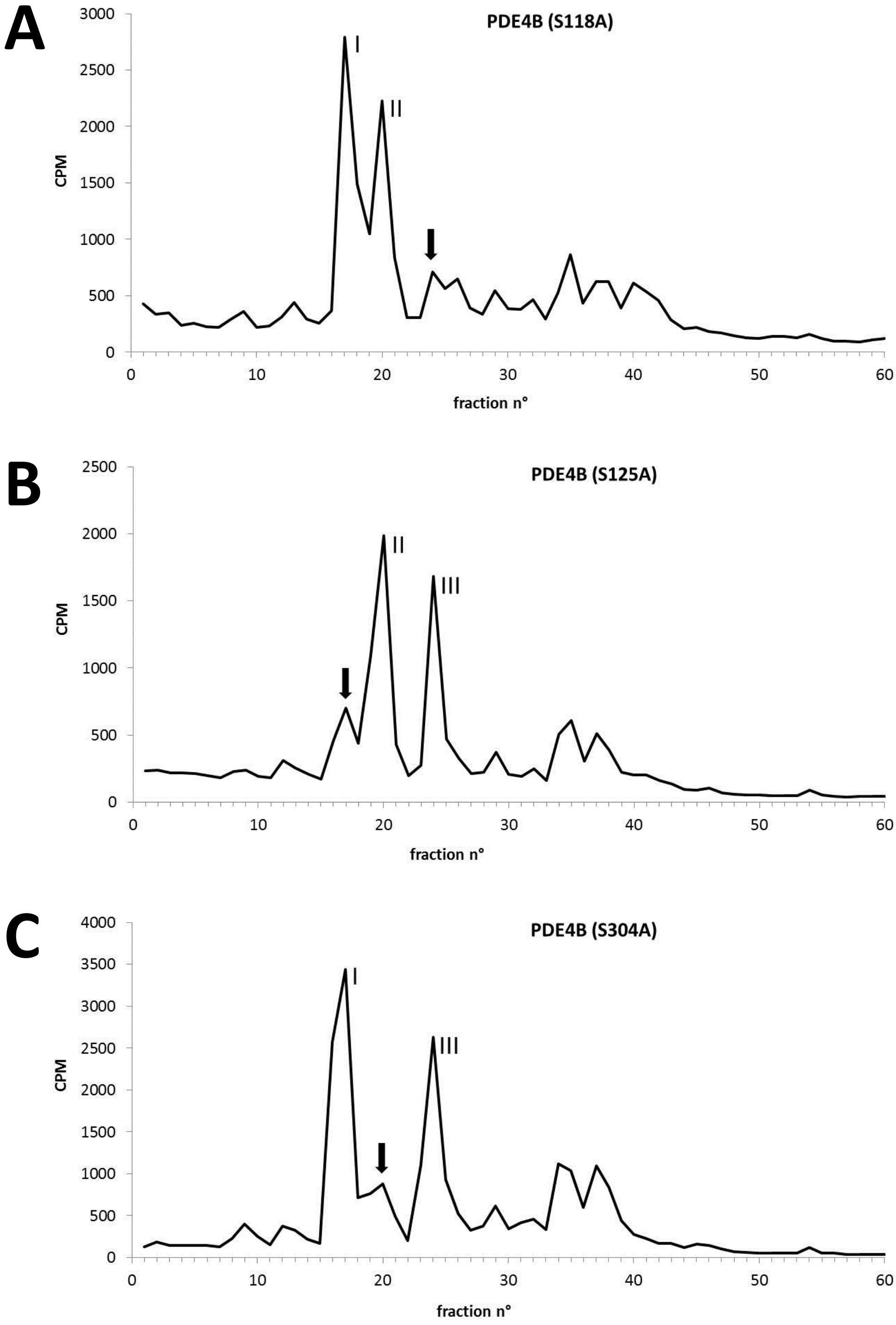

Supplementary Fig. 5: Uncropped scans of the blots, autoradiograms and Coomassie Blue-stained gels used to generate the main manuscript figures. Full scan images are shown in which the migration of the protein of interest is indicated by an arrow. The migration of the molecular weight markers relative to the protein of interest is indicated in the corresponding figures of the main manuscript.

Main manuscript Figure 1B

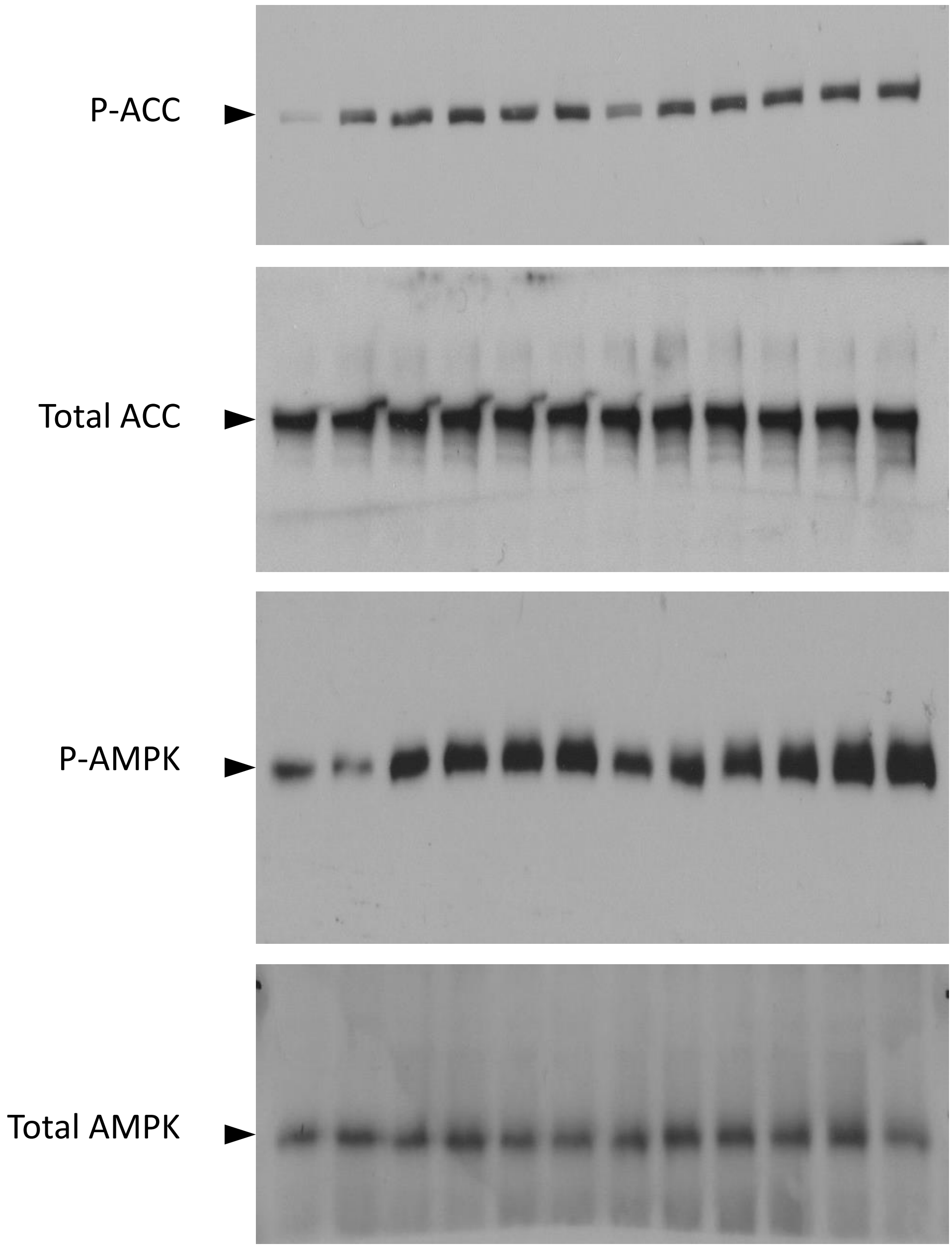

Main manuscript Figure 1B

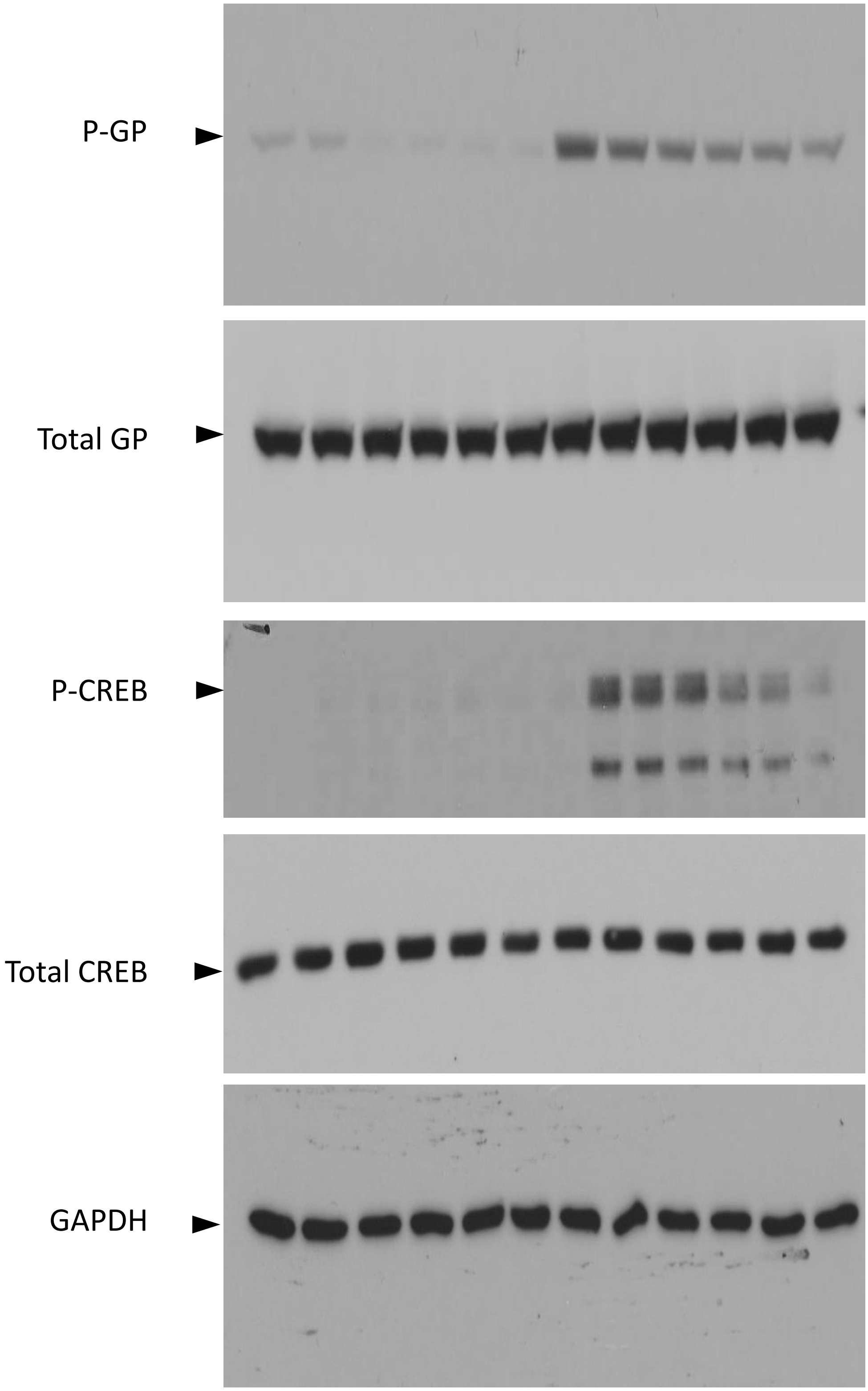

Main manuscript Figure 1D

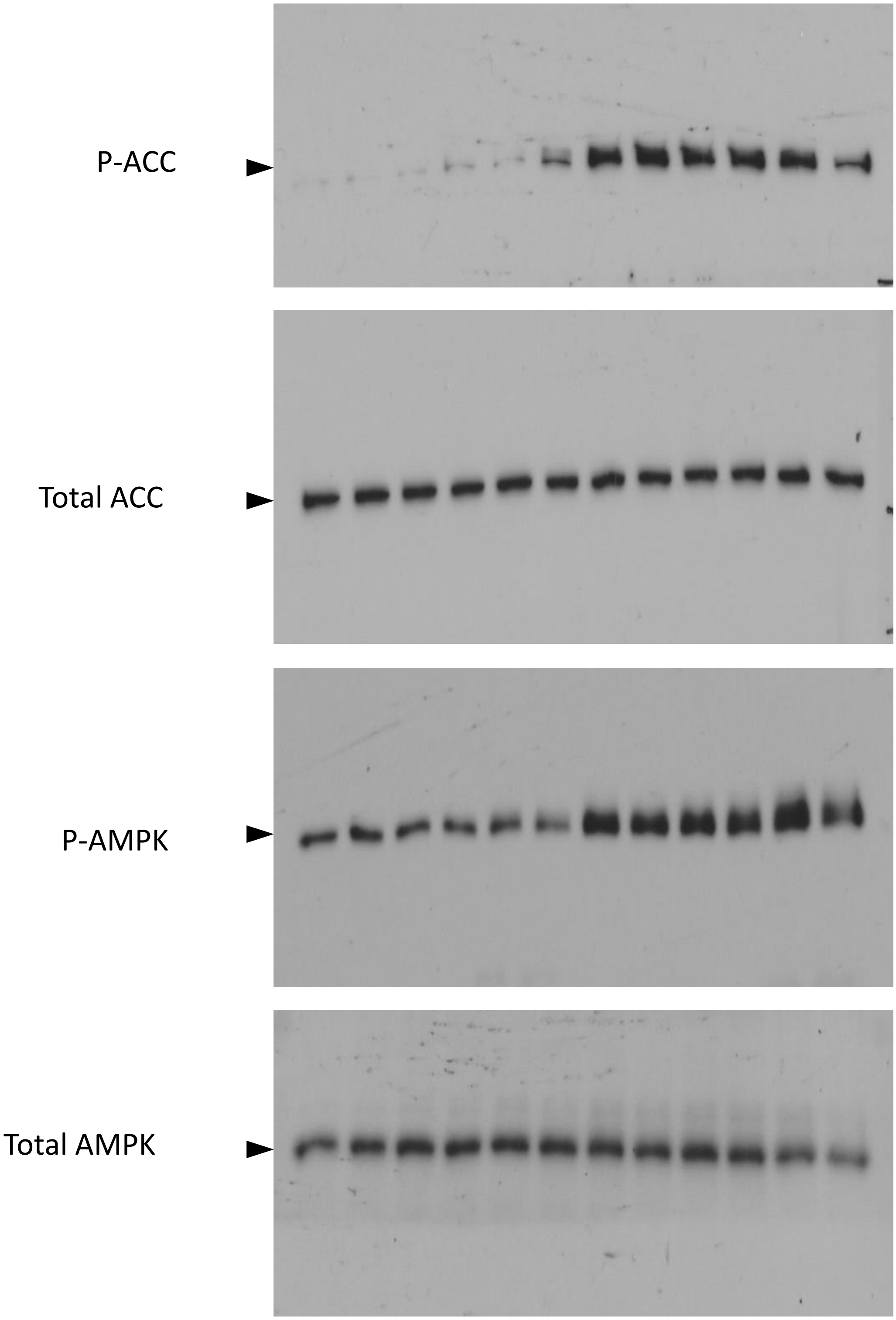

Main manuscript Figure 1D

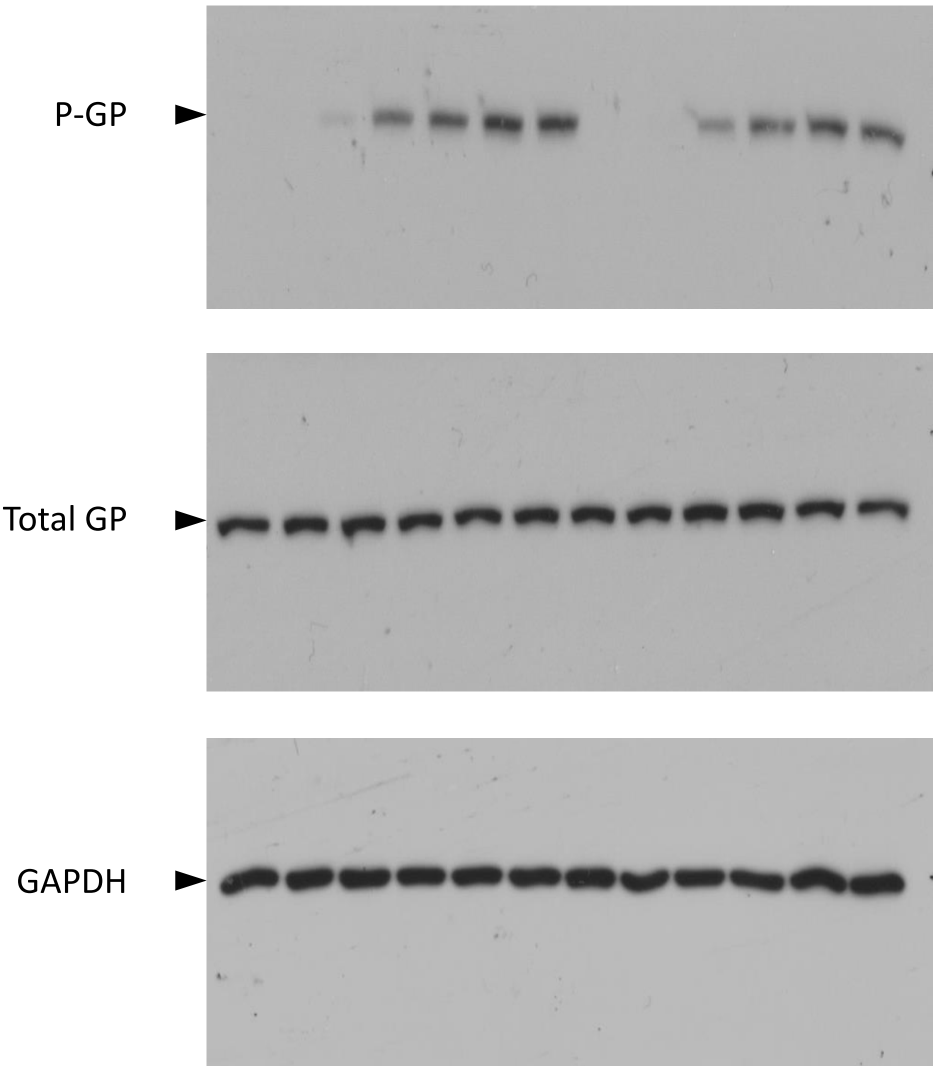

Main manuscript Figure 3A – Wild-type

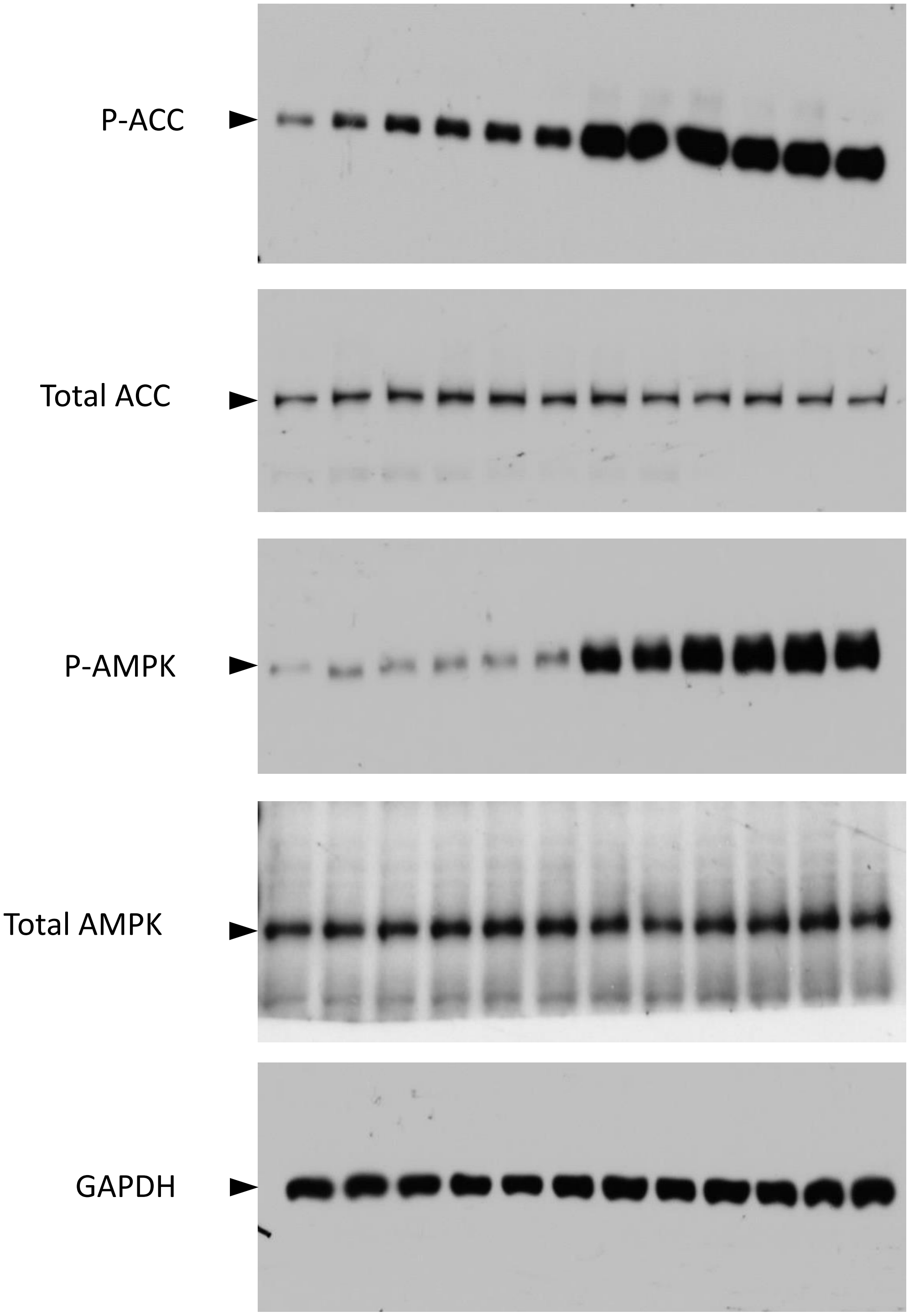

Main manuscript Figure 3A – AMPK $\alpha_1^{-/-}$ $\alpha_2^{LS-/-}$

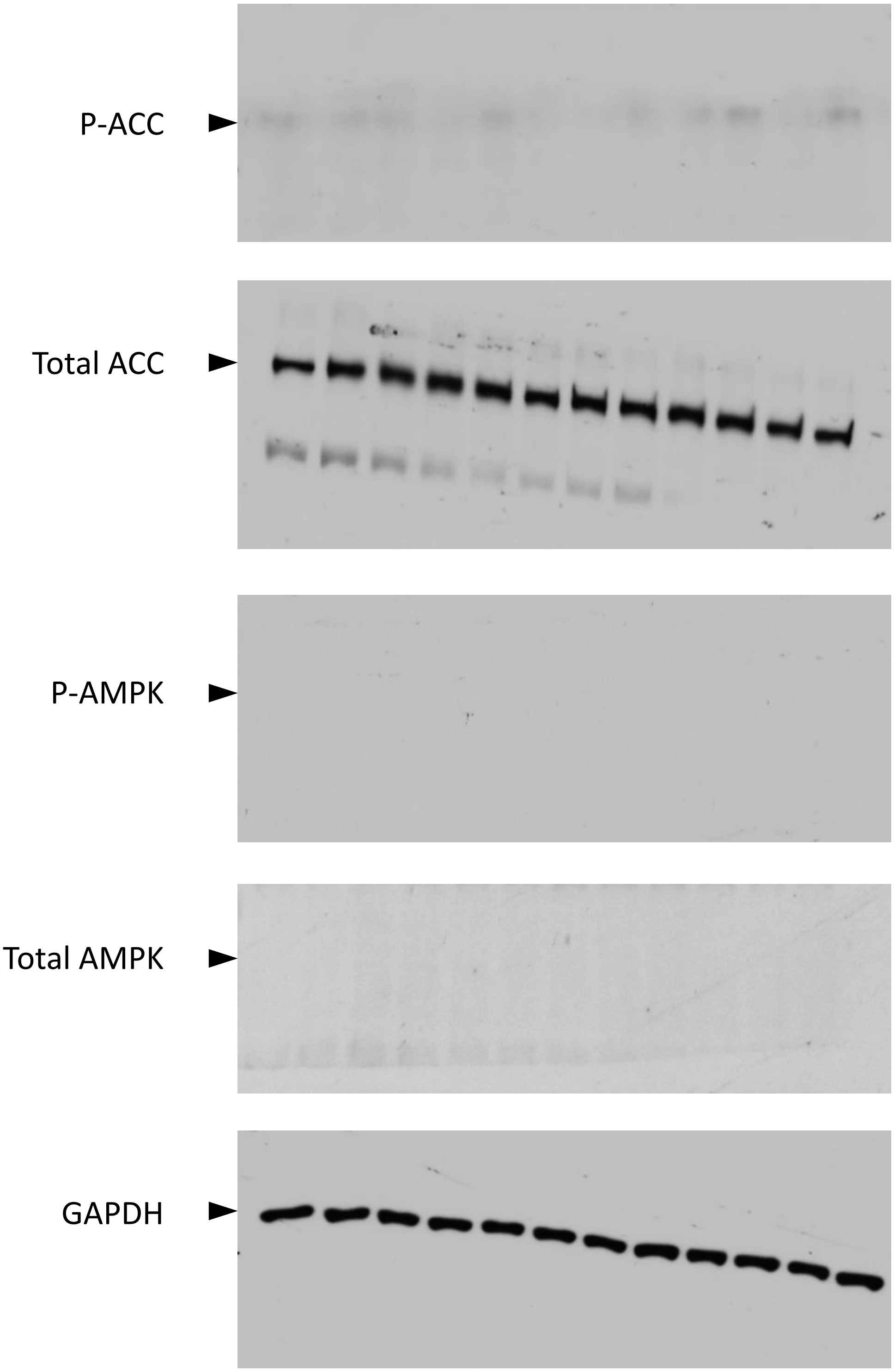

Main manuscript Figure 5A

Autoradiogram

^{32}P-PDE4B

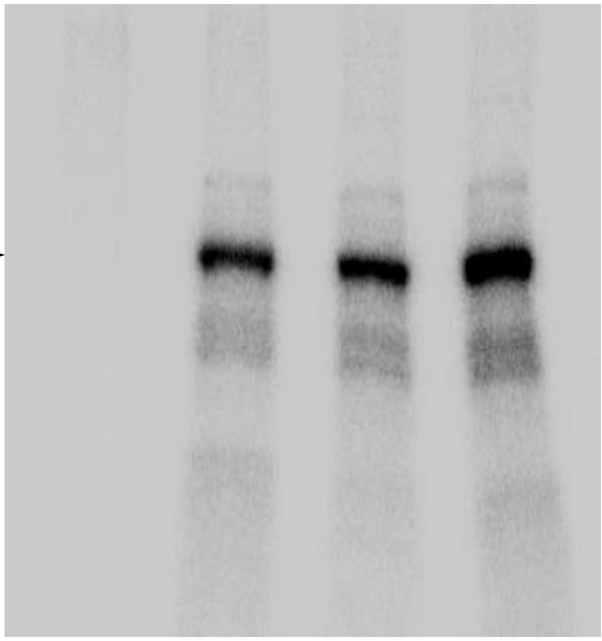

Coomassie stain

Total PDE4B

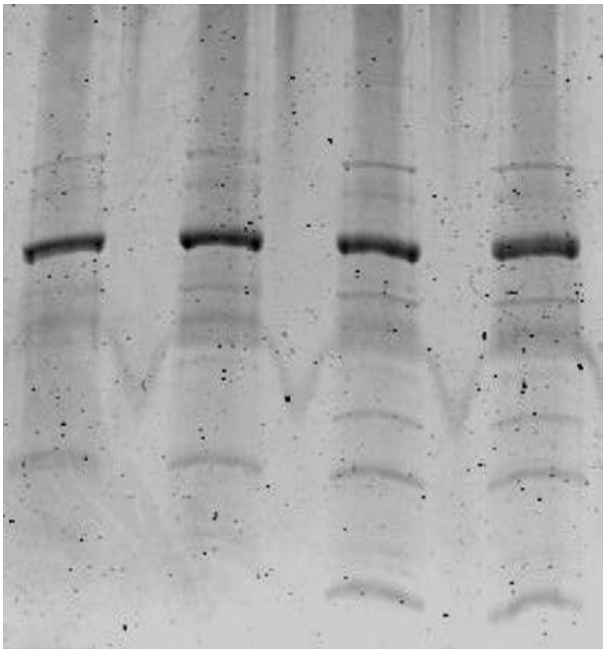

Main manuscript Figure 5C

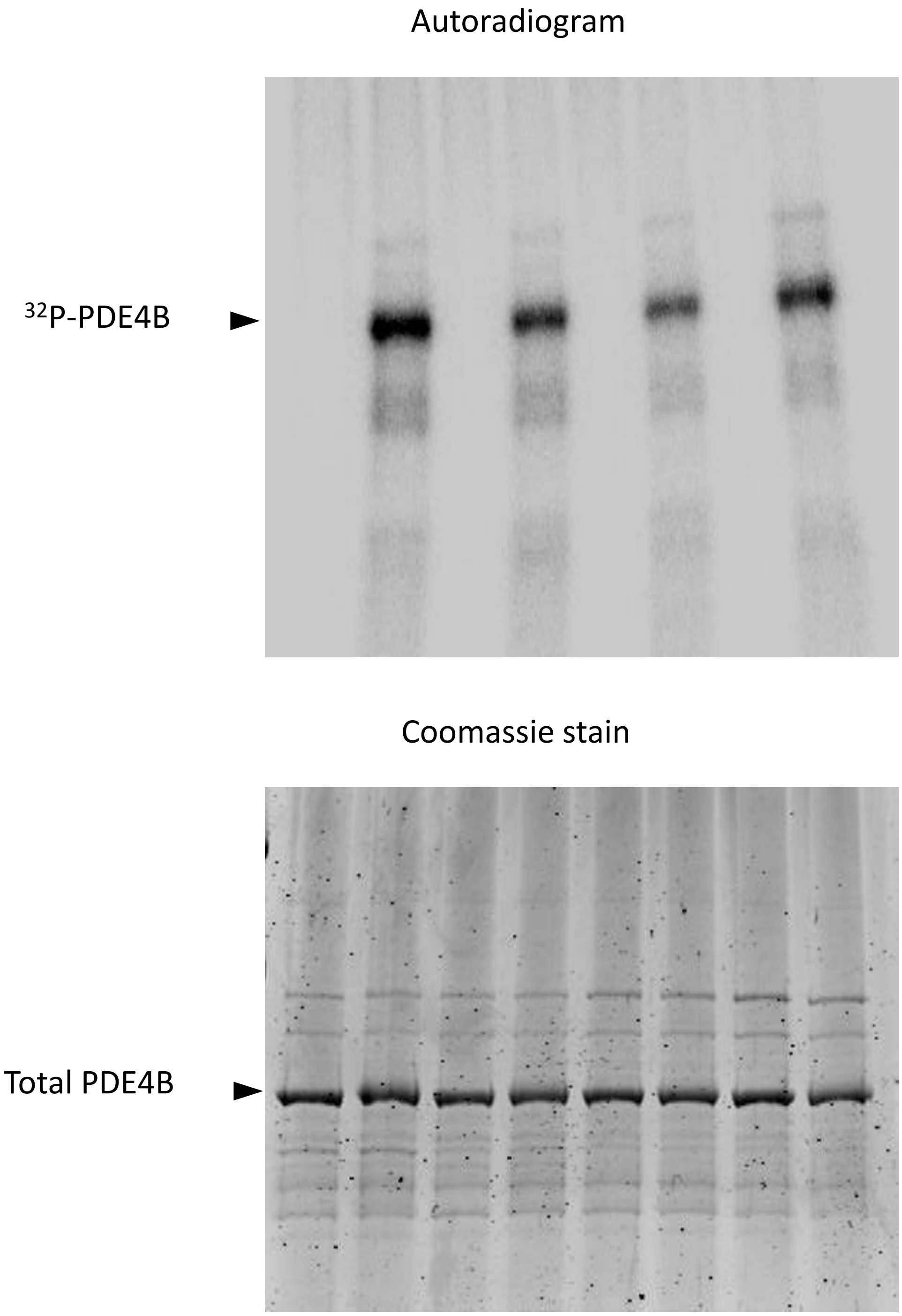

Main manuscript Figure 6A

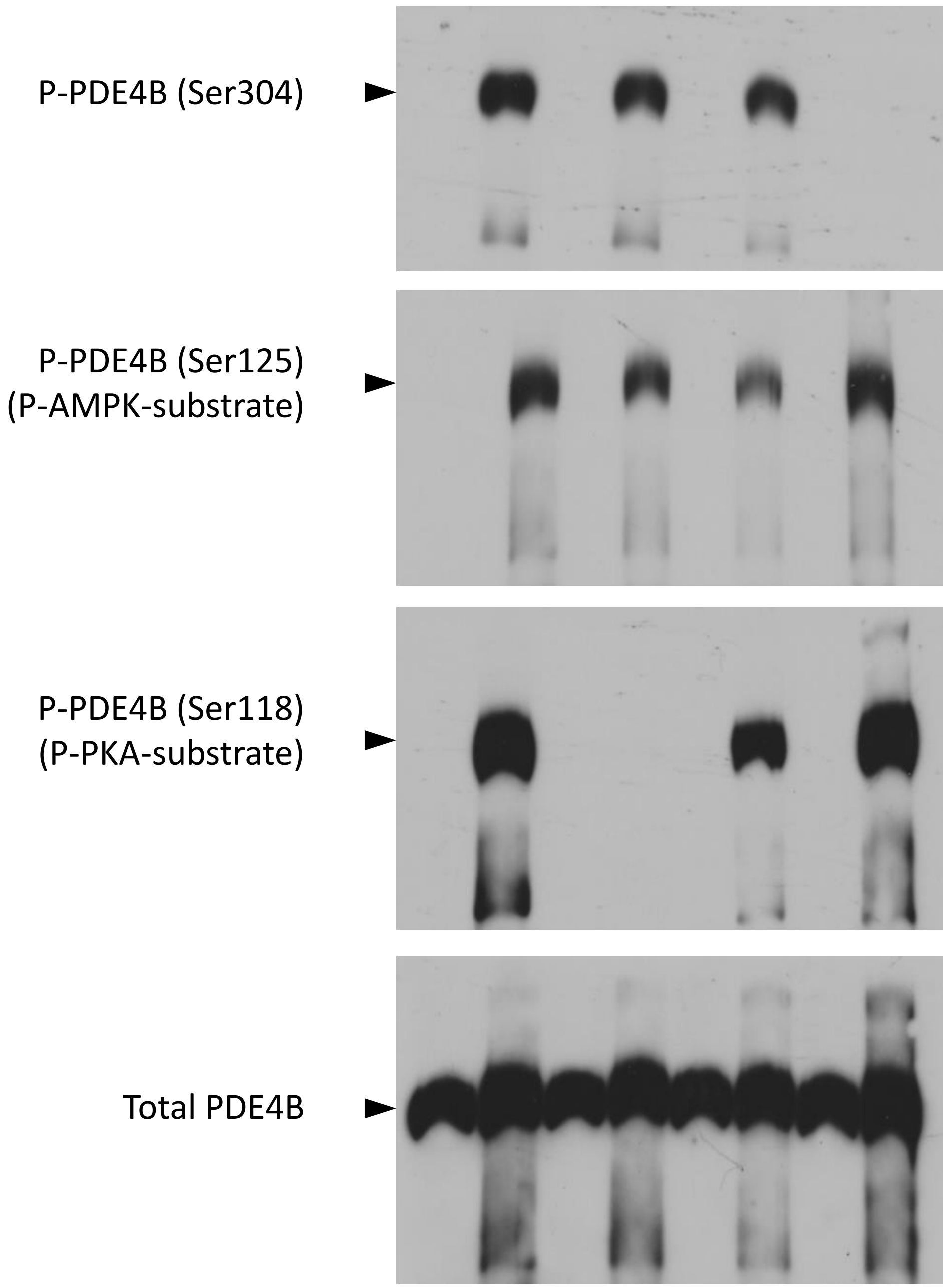

Main manuscript Figure 6B

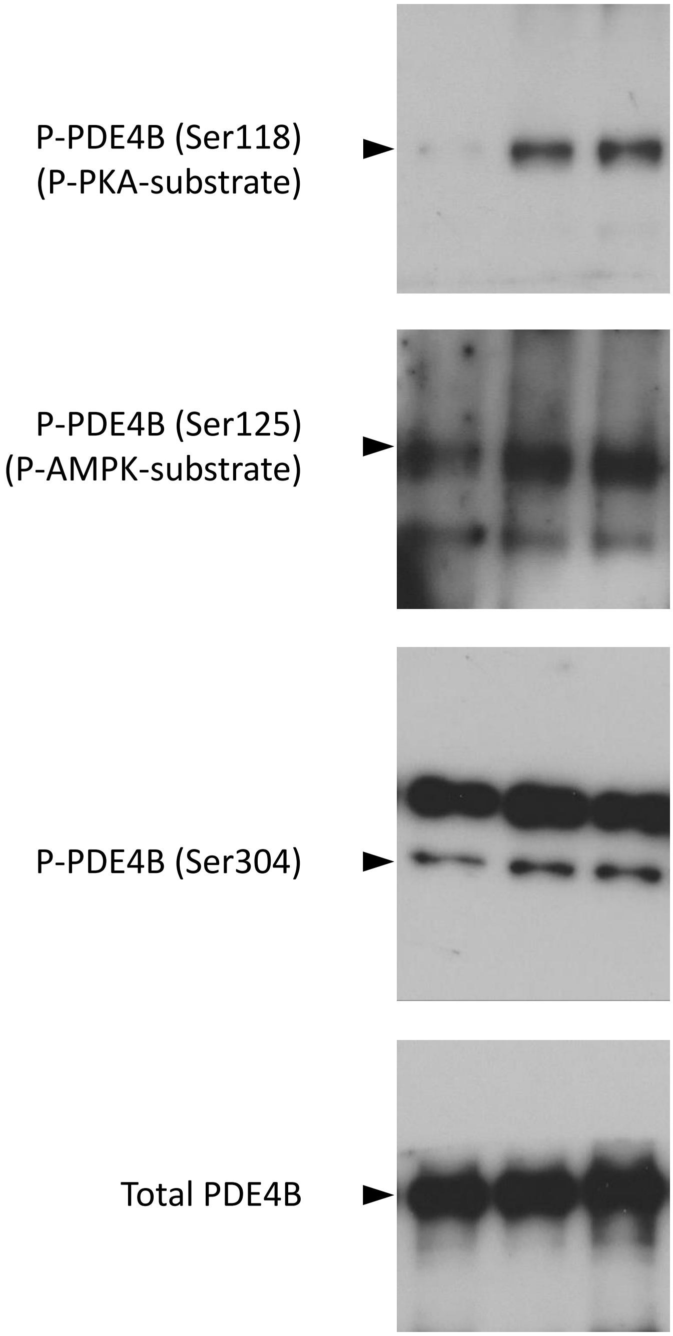

Main manuscript Figure 6C

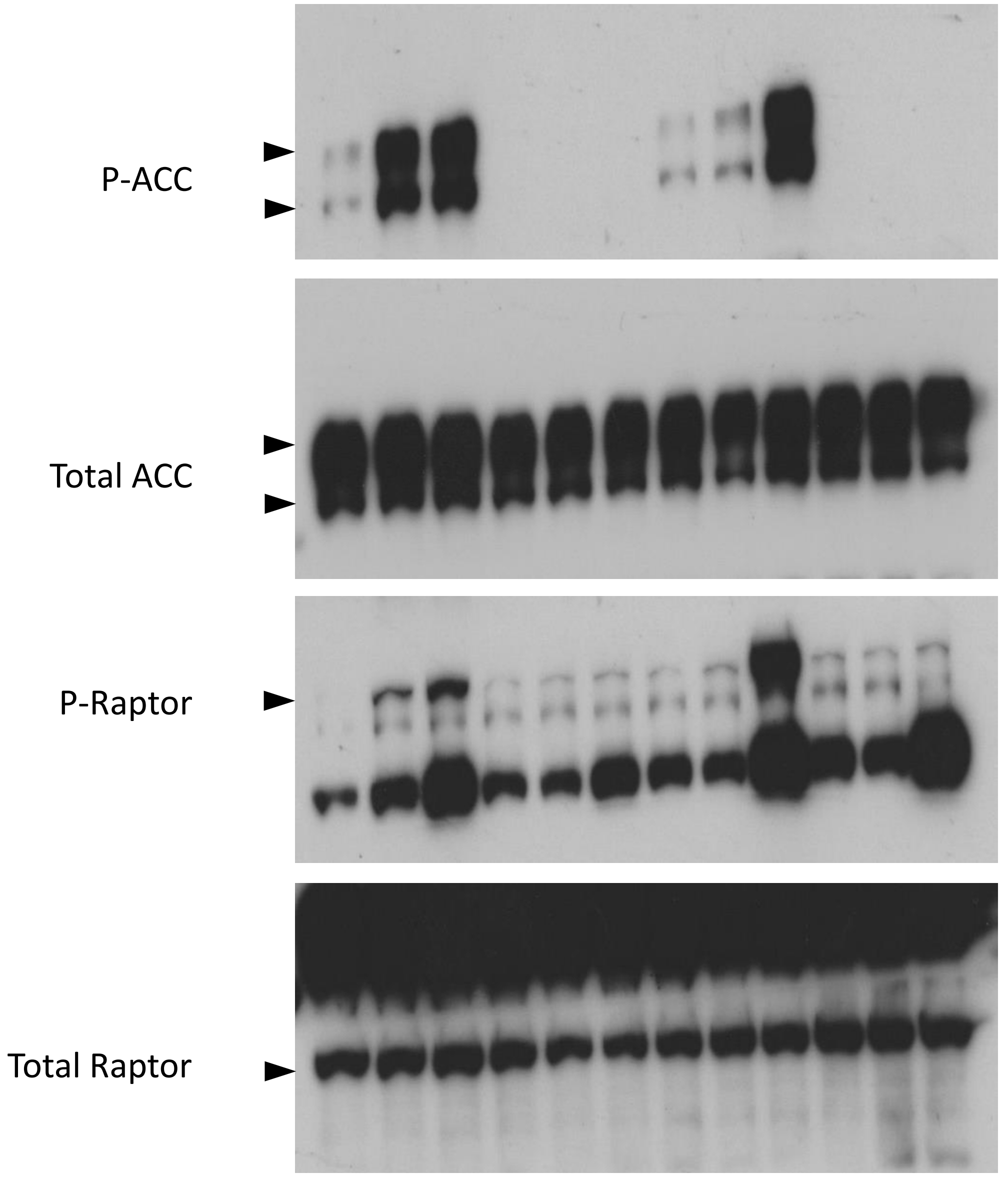

Main manuscript Figure 6C

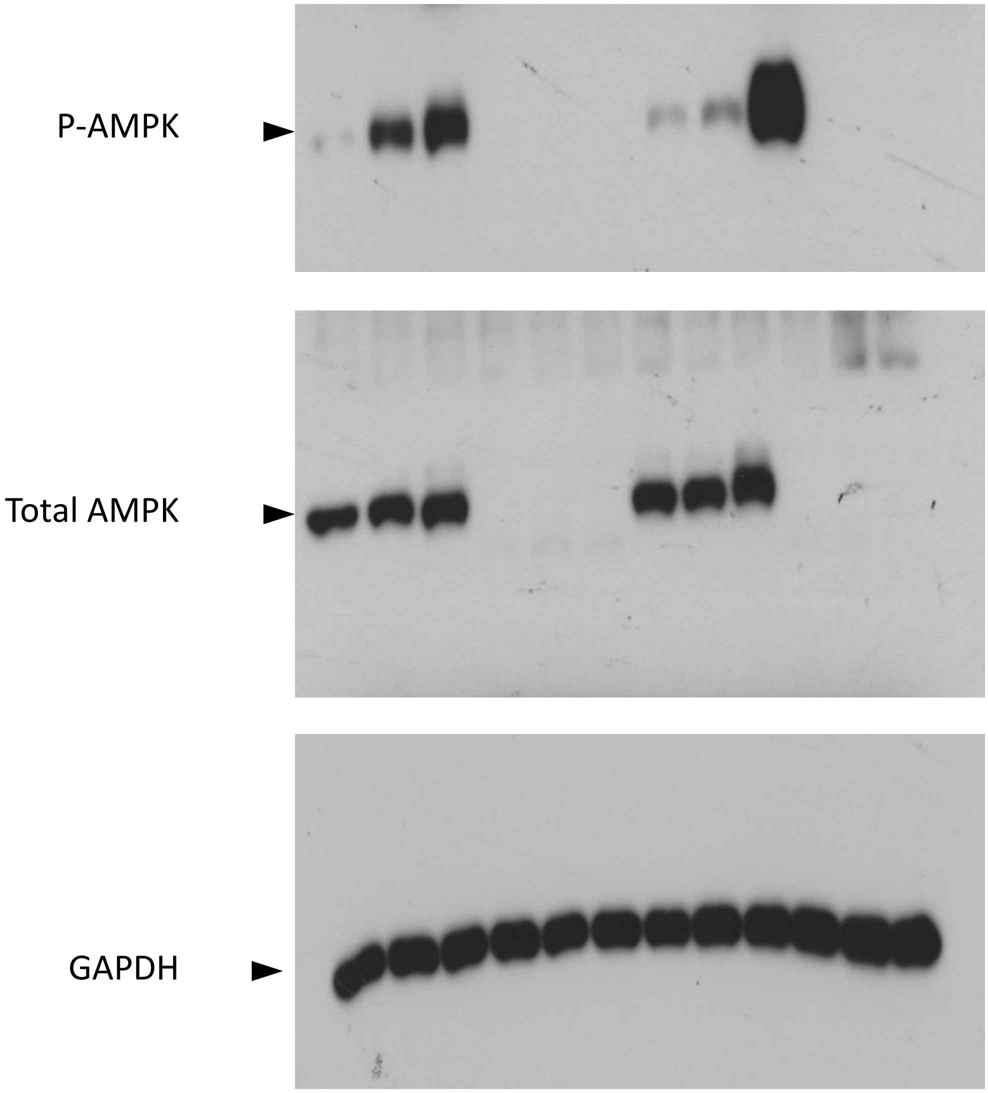

Main manuscript Figure 6C – Wild-type

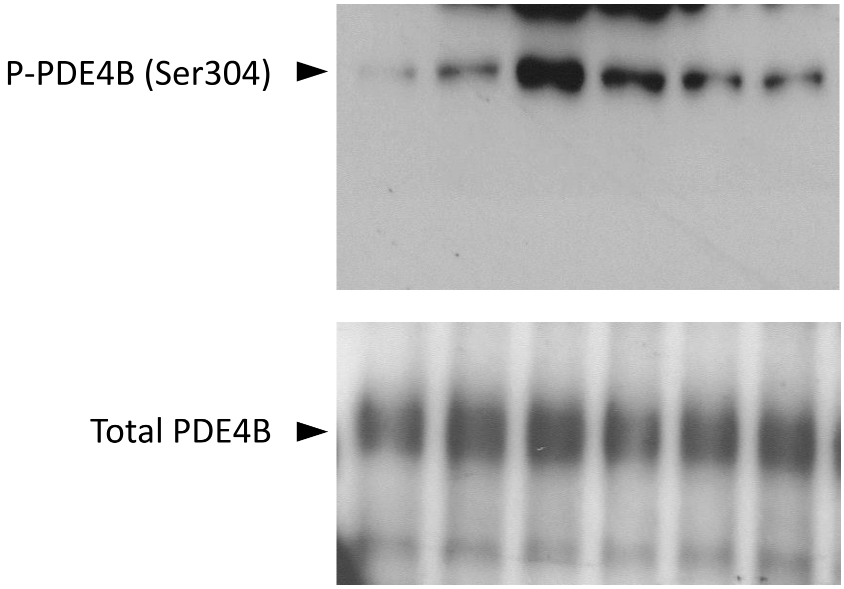

Main manuscript Figure 6C – AMPK $\alpha_1^{-/-}$ $\alpha_2^{LS-/-}$

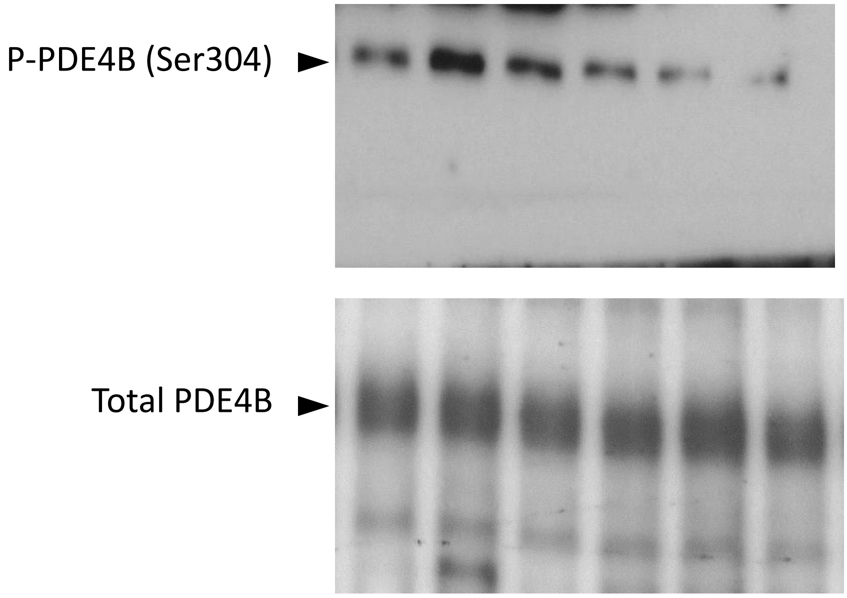

Supplement: Supplementary Information — Supplementary Figures 1-5 [file ncomms10856-s1.pdf]
